# Structural diversity of *Burkholderia pseudomallei* lipopolysaccharides affects innate immune signaling

**DOI:** 10.1371/journal.pntd.0005571

**Published:** 2017-04-28

**Authors:** Michael H. Norris, Herbert P. Schweizer, Apichai Tuanyok

**Affiliations:** 1 Department of Infectious Diseases and Pathology, College of Veterinary Medicine, University of Florida, Gainesville, Florida, United States of America; 2 Emerging Pathogens Institute, University of Florida, Gainesville, Florida, United States of America; 3 Department of Molecular Genetics and Microbiology, College of Medicine, University of Florida, Gainesville, Florida, United States of America; Mahidol University, THAILAND

## Abstract

*Burkholderia pseudomallei* (*Bp*) causes the disease melioidosis. The main cause of mortality in this disease is septic shock triggered by the host responding to lipopolysaccharide (LPS) components of the Gram-negative outer membrane. *Bp* LPS is thought to be a weak inducer of the host immune system. LPS from several strains of *Bp* were purified and their ability to induce the inflammatory mediators TNF-α and iNOS in murine macrophages at low concentrations was investigated. Innate and adaptive immunity qPCR arrays were used to profile expression patterns of 84 gene targets in response to the different LPS types. Additional qPCR validation confirmed large differences in macrophage response. LPS from a high-virulence serotype B strain 576a and a virulent rough central nervous system tropic strain MSHR435 greatly induced the innate immune response indicating that the immunopathogenesis of these strains is different than in infections with strains similar to the prototype strain 1026b. The accumulation of autophagic vesicles was also increased in macrophages challenged with highly immunogenic *Bp* LPS. Gene induction and concomitant cytokine secretion profiles of human PBMCs in response to the various LPS were also investigated. MALDI-TOF/TOF was used to probe the lipid A portions of the LPS, indicating substantial structural differences that likely play a role in host response to LPS. These findings add to the evolving knowledge of host-response to bacterial LPS, which can be used to better understand septic shock in melioidosis patients and in the rational design of vaccines.

## Author summary

Not only does *Burkholderia pseudomallei* pose a public health challenge in Southeast Asia and northern Australia but in the United States (US) *Bp* is listed as a Tier-1 select agent. Tier 1 organisms have the potential to pose a severe threat to US public health and safety and policy makers mandated development of vaccines and therapeutics for them, including *Bp*. Mortality is most often caused by septic shock. Understanding the mechanisms by which a variety of *Bp* strains cause septic shock can reduce mortality associated with melioidosis but also allow for the rational design of effective vaccines.

## Introduction

*Burkholderia pseudomallei* (*Bp*), is typically found in soil and water environments in the tropics [2]. In Thailand, very high levels of the bacterium are often found in the pooled surface waters of rice paddies [3] coinciding with high disease and seropositivity rates in rice farmers. Typically there is a regional hospital away from the individuals home and by the time they arrive at the hospitals, the disease has progressed acutely, resulting in a high mortality rate of 40.5% [4]. Seventy- nine percent of individuals diagnosed as having melioidosis are farmers and nearly 70% of those are patients that have type 2 diabetes mellitus that is poorly controlled or undiagnosed [5]. Not only does *Bp* pose a public health challenge in Southeast Asia and northern Australia, but a recent study estimated that *Bp* prevalence and concomitant melioidosis burden are considerably more widespread than previously thought. In terms of mortality, melioidosis may actually be one of the dominant tropical killer diseases. *Bp* is also considered a biothreat agent and in the US is listed as a Tier-1 Select Agent with a mandate to develop medical countermeasures that include therapeutics and vaccines [1].

When *Bp* cells come into contact with host cells, a plethora of host modifications occurs in response to bacterial infiltration and virulence factor expression eventually leading to a disease state. Within-host, *Bp* can infect, invade most tissues, and replicate inside the cytoplasm of many cell types [6–11]. To accomplish this feat, *Bp* attaches to the host cell via an unknown mechanism, causing actin rearrangement and inducing bacterial phagocytosis [12]. Once attached, *Bp* utilizes the *Burkholderia secretion apparatus* (*Bsa*, one of three genomically encoded type 3 secretion systems, T3SS) to secrete the effector BopE through the cytoplasmic membrane and this event is required for full virulence [13, 14]. BopE is a guanine nucleotide exchange factor for Rho family GTPases that undermines the surrounding cytoskeletal framework, inducing invasion of the host-cell by bacterial phagocytosis [15]. The *Bsa* effector protein BopA then allows vesicular escape before phagosomal degradation of *Bp* occurs [16]. Once inside the cytoplasm the well characterized *Bp-*intracellular motility protein A, BimA, polymerizes host-cell actin [17]. The polar actin “tail” allows intracellular movement and eventually leads to formation of membrane protrusions [18]. The membrane protrusions allow *Bp* to seek out uninfected cells and begin the cycle anew without transitioning into the extracellular milieu. During the infectious process an infected cell may fuse with viable neighboring host cells and this is attributed to the main *Bp* type 6 secretion system (T6SS-1) [19]. The formation of multi-nucleated giant cells (MNGCs) has been observed *in vitro* and *in vivo* and is a major hallmark of *Bp* infection [18, 20]. The bacteria replicate concurrently with the production of several known and unknown virulence factors causing death of the host-cell. Due to high levels of genetic diversity and a flexible genome [21, 22] not all virulence factors are universal; resulting in a variety of bacterial virulence factors expressed *in vivo* depending on the genetic background. For example, many strains in Australia have the *B*. *thailandensis* (*Bt*)*–*like flagellar cluster (BTFC), a lateral flagellar system in addition to the polar flagellar system, while a majority of strains from Thailand and other countries in Asia have the *Yersinia*-like fimbriae (YLF) cluster at the same genomic location [23, 24]. Additionally, the diversity of LPS profiles between different *Bp* strains has been demonstrated with discovery by the investigators of multiple LPS types [25].

When *Bp* invades the host with such an assortment of virulence factors, the resulting immune response to bacterial pathogen associated molecular patterns (PAMPs) attempts to clear the bacteria, causing host organ and tissue damage associated with melioidosis. Inflammation induced by *Bp* infection is a major player in the immunopathogenesis of melioidosis. The end result is runaway inflammation and endotoxic shock, the primary cause of death in melioidosis. Thus, a major bacterial mediator of immunopathogenesis is the LPS of *Bp*. Previously published data have shown that pro-inflammatory cytokine levels induced by *Bt* type A LPS, through TLR4, were higher than LPS from *Bp*, due to structural differences in the lipid A portion of LPS [26]. A more contentious finding is that *Bp* LPS can signal through TLR2 and TLR4 [27–30]. There have been many claims that observed TLR2 signaling is because of lipoprotein contamination of LPS preparations. Recently this has been shown not to be the case since *Bp* strain 1026b LPS can signal through TLR2 and TLR4 in human cells but only weakly in mouse cells after 24 h of exposure [29]. This is not farfetched, tri-acylated lipoproteins can be chaperoned by LPS binding protein (LBP) and CD14 to signal through TLR2 heterodimers [31–33] and small amounts of tri-acylated and more tetra-acylated LPS have been identified in *Bp* LPS. Efficient signaling through both TLR2 and TLR4 can result in synergistic production of pro-inflammatory cytokines [34, 35].

Amongst *Bp*, three types of LPS (type A, B, and B2) have been identified [25] with *B*. *mallei* (*Bm)* representing a fourth, type A with a variable acetylation pattern that we have termed type A variant (A_V_). In *Bp* it is predicted that ~97% of strains in Thailand and ~85% of strains in Australia have the type A LPS. A few clinical isolates from Thailand have type B LPS and many times more in Australia and Papua New Guinea have type B and B2. It is unknown what LPS types predominate in other areas of the world including South Asia, the Middle East, Africa, and the Americas but thus far virulent type B strains make up a majority of strains isolated in Madagascar [36]. Antibodies to one type are not cross-reactive between A and B serotypes as observed via Western blot [25] due to structural differences of the O-antigen. The type A LPS is a repeating subunit of glucose and talose [37]. The type B LPS on the other hand is made of repeating subunits of the xylose-rhamnose-rhamnose-rhamnose-rhamnose-galactose polysaccharide (preliminary data is presented within this manuscript). Type B and B2 antibodies are cross-reactive and recognize similar epitopes of different LPS structures that are not shared with Type A LPS [38]. Type B2 is a repeating polysaccharide of rhamnose-rhamnose-galactose. Several strains of near neighbor species including *B*. *ubonensis* and *Bt–*like species, have the type B or B2 LPS, respectively [39]. *B*. *mallei* (*Bm*) on the other hand has a variant type A LPS, which is missing the 4-*O* acetylation of the talose that may affect epitope recognition and antibody specificity [40].

Within *Bp* there is great genetic diversity. This diversity extends to the highly immunogenic LPS molecule. We sought to investigate if structural diversity of the polysaccharide portion of LPS was indicative of further differences in the lipid A side and acyl chain modifications in type strains of research interest. In this work, the glycosyl composition of the uncharacterized type B, B2, and rough LPS verified *O*-antigen structural differences. Differential induction of innate immunity by the LPS from the *Bp* type strains was investigated in murine macrophages and verified in human donated peripheral blood mononuclear cells (PBMCs). Large differences in pathway gene activation and cytokine secretion amongst the *Bp* LPS were found. The cytokines produced by the innate response are known to play an important role in shaping an effective adaptive response [41] and so the cytokine environment produced in response to the *Bp* LPS types was characterized. *Bp* lipid A masses from the type strains were then elucidated by MALDI-TOF/TOF. Structural diversity in the lipid A region may allow for modulation of the innate immune response and adaptive immune responses. Taken together, this work has ramifications on vaccine design due to diversity of the *O*-antigen and lipid A of *Bp* LPS and enhances our understanding of the host inflammatory response to pathogenic bacterial LPS with potential impact on virulence and sepsis research.

## Methods

### Bacterial strains, cell lines and culture conditions

All select agent work was carried out in a CDC/USDA Tier 1 approved BSL-3 facility at the University of Florida following Tier 1 regulations. All protocols were approved by the Institutional Biosafety Committee prior to implementation. *Bp* strains (CDC/USDA registered in house bacterial inventory) were grown on Lennox broth (5 g/L NaCl)(LB, Fisher BioReagents) or Tryptic Soy Agar (Becton Dickinson) and grown at 37°C. LB broth was used for liquid growth of all strains. LB supplemented with 1,000 μg/mL kanamycin (Km, Fisher Scientific) was used for selection of mutants in *Bp* strains. *E*. *coli* strain NEB5α was used as a cloning strain (New England Biolabs). Selection of Km resistant *E*. *coli* strains was performed on LB medium with 35 μg/mL Km. Select Agent excluded strain Bp82 [42] and its derivatives (H. Schweizer Lab) were grown on LB or TSA with 0.6 mM adenine (Amresco). The Bp82 and 576a Δ*wcb* capsule mutants were created using previously published methods [42–44] [see Supplemental Materials and Methods. Mouse cell lines RAW264.7 and L929 (American Type Culture Collection, ATCC) were grown in Dulbecco’s Modified Eagle Medium (DMEM)-high glucose+L-glutamine (HyClone) with 10% FBS (HyClone) in 5% CO_2_ at 37°C. All plastic ware was Corningware with CellBIND surface. Culturing cells was carried out essentially as described previously [10, 43, 45, 46].

Details on bacterial mutant generation can be found in the S1 Methods.

### LPS isolation

A modified hot-phenol extraction was utilized to extract LPS from select agent excluded and select agent *Bp*. This was done essentially as described [47] but with modifications included for BSL-3 activities. Each bacterial strain was grown on 8–10 plates of TSA or LB-agar for 48–72 h. Bacterial lawns were flooded with TBS and scraped off using a plate spreader. The bacterial suspensions were distributed into 2 mL gasketed microcentrifuge tubes and heat-killed at 110^°^C for 15 minutes. Phenol was added to the lysed solution to a final concentration of 50% and 10% of the resulting mixture was plated on TSA to ensure sterility. Upon verification of sterility, the samples were moved to the BSL-2 and the protocol was continued as described. Samples were dialyzed using tubing with 12–14 kDa molecular weight cutoff against distilled water for 3–5 days until free of phenol. Samples from both phenol and aqueous phases were checked for presence of LPS by silver staining with 1026b LPS partitioning to the phenol phase, 576a and MSHR840 partitioning to the aqueous phase while LPS from MSHR435 had two different LPS species one in each of the phenol and aqueous phases. The phases of each LPS isolation were combined, lyophilized, treated with DNase I for 2 h, RNase H for 2 h, and Proteinase K overnight, then further purified as previously described [47]. After the final lyophilization, dry weights were determined and the residues resuspended in 2 mL of endotoxin free water. The samples were diluted and endotoxin units were measured using the Pierce LAL assay according to the manufacturer’s instructions. Samples were weighed and endotoxin units per unit weight were determined in duplicate and compared to S-form LPS from *Salmonella minnesota* (Hycult) that was quality controlled for undetectable TLR4 independent activity.

### Glycosyl composition analysis

Glycosyl composition analysis was performed by combined gas chromatography/mass spectrometry (GC/MS) of the per-O-trimethylsilyl (TMS) derivatives of the monosaccharide methyl glycosides produced from the sample by acidic methanolysis as described previously [48]. Briefly, the samples were heated with methanolic HCl in a sealed screw-top glass test tube for 18 h at 80°C. After cooling and removal of the solvent under a stream of nitrogen, the samples were treated with a mixture of methanol, pyridine, and acetic anhydride for 30 min. The solvents were evaporated, and the samples were derivatized with Sylon HTP (Sigma) at 80°C for 30 min. GC/MS analysis of the TMS methyl glycosides was performed on an Agilent 7890A GC interfaced to a 5975C MSD, using an Supelco Equity-1 fused silica capillary column (30 m × 0.25 mm ID).

### RAW264.7 TNF-α secretion assays

TNF-α bioassays were carried out by exposing RAW264.7 macrophages (ATCC) to the indicated concentration of purified LPS for 6 and 24 h in biological triplicate as previously described [49]. The supernatant was then mixed with actinomycin D to a final concentration of 8 μg/mL and incubated with L929 mouse fibroblasts (ATCC) for 20 h in triplicate. Two-fold dilutions of recombinant mouse TNF-α (BD Fisher) were also used to treat L929 monolayers for 20 h. The media were removed and the monolayers washed once with 1xPBS and then fixed for 30 min with 4% paraformaldehyde in PBS. The fixative was then removed and 50 μL of a 0.5% (w/v) crystal violet solution was added to each well and allowed to stain for 10 min. The stain was removed and washed with deionized water twice. The crystal violet was solubilized with 200 μL of 1% (w/v) SDS and the A_540nm_ read using a plate reader. % cytotoxicity was expressed as % cytotoxicity = 100 x [1-(OD_noRx_-OD_Rx_/OD_noRx_)]. A standard curve was generated from L929 cells treated with the two-fold dilutions of known quantities of purified TNF-α. The standard curve was used to calculate amounts of secreted TNF-α in the supernatants of the LPS treated RAW264.7 macrophages.

### RAW264.7 nitric oxide assay

The NO assays were preformed essentially as above but the supernatant from LPS treated RAW264.7 macrophages was used to determine the total amount of NO secreted into the media using the Thermo Scientific Total Nitric Oxide Assay Kit according to the manufacturer’s instructions. RAW264.7 macrophages were seeded in 24-well Corning CellBIND plates and allowed to attach overnight. In the morning the LPS samples were diluted to the indicated concentrations in DMEM and placed on the monolayers. At 6 and 24 h the media was removed and NO measured. This nitric oxide assay measured amount of nitrite (NO_2_-) and nitrate (NO_3_-) released into the cell culture media. In the assay, the enzyme nitrate reductase converts nitrate to nitrite. Nitrite in each sample supernatant is then detected as a colored azo dye product of the Griess reaction that absorbs visible light at 540 nm. Total nitric oxide (NO) contributed by nitrate and nitrite release in response to LPS treatment was measured as nitrite after converting all nitrate to nitrite. Absorbance values were compared to a Nitrate Standard curve. Experiments were carried out in biological triplicate.

### Gene expression analysis of innate and adaptive immunity in mouse macrophages in response to purified *Bp* type A and type B LPS

RAW264.7 macrophages were seeded in 24-well CellBIND plates and allowed to attach overnight. LPS isolated from a 1026b capsule mutant and a 576a capsule mutant was used to treat RAW264.7 macrophages at 10 ng/ml for 2 h in triplicate. At 2 h post-treatment the media was removed and the monolayers incubated in RNAprotect Cell Reagent solution. The cells were stored at -80°C until further use. RNA was isolated using the RNeasy Mini Kit (QIAGEN) according to the manufacturer’s protocol and mRNA integrity and concentration was determined by agarose gel visualization and UV absorbance measurements. cDNA was synthesized using the Superscript III First-Strand Synthesis System (Thermo Fisher) according to the manufacturers protocols. Innate and adaptive mouse RT^2^ Profiler qPCR arrays were obtained from QIAGEN. These arrays contain RT controls, gDNA contamination controls, multiple housekeeping genes, and negative controls. The cDNA was diluted 1:10 and the arrays were run on a Bio-Rad CFX-96 using SsoAdvanced Universal SYBR Green Supermix (Bio-Rad). This experiment was carried out in biological triplicate using three arrays each for 1026b, 576a, and untreated macrophage cDNA. Cycle threshold values were exported and pathway analysis was carried out through the QIAGEN GeneGlobe portal.

### Fluorescence microscopy and LC3 puncta assay

RAW264.7 murine macrophages were seeded in Millipore EZ Slides and allowed to attach overnight. To allow for proper induction of autophagy the monolayers were treated with 10 ng/mL of the various LPS for 16 h in triplicate. At the end of 16 h, the media were removed and the monolayers washed with 4% (w/v) paraformaldehyde in PBS for 15 min. Fixed cells were washed twice with PBS then permeabilized by incubation with 0.2% (v/v) Triton X-100 in PBS for 10 min. Cells were then washed in PBS three times for 5 min each. Cells were blocked with 1% (w/v) BSA for 30 min at room temperature then mouse anti-LC3 mAb (MBL International) in 1% BSA (w/v) in PBS for 1 h. This antibody recognizes the pre- and post- membrane insertion LC3, LC3-I (diffuse cytosolic) and LC3-II (membrane bound puncta) respectively. Cells were then washed with PBS three times for 5 min each and incubated with goat anti-mouse IgG conjugated with Alexa Fluor 488 in 1% BSA/PBS for 1 h at room temperature. Cells were washed with PBS three times for 5 min each then mounted in ProLong Gold Antifade Reagent with DAPI and allowed to cure overnight. Images were captured using a Leica DM2500 fluorescent microscope. Images were processed, deconvoluted, and analyzed using ImageJ. Three images were taken of each replicate and masked by signal intensity. GFP puncta equating to high levels of membrane bound LC3 (LC3-II) were counted. Nuclei associated with more than 10 puncta were considered LC3 positive cells. At least 50 cells per image were counted.

### Cell infection LC3 assays

The infection assay was performed by diluting *Bp* strains grown in LB medium overnight at 37^°^C in PBS and then the desired CFU/mL in Dulbecco’s Modified Eagle Medium (DMEM). The dilutions were used to infect RAW264.7 macrophages in 8-well Millicell EZ Slides (Millipore) at an MOI of 10:1. At 6 h post infection the bacteria-containing medium was removed and the monolayers were washed 3 times with pre-warmed PBS. Monolayers were fixed with 4% paraformaldehyde in PBS for 10 min and 95% ethanol for 5 min. Then washed in PBS. The assay was carried out in biological triplicate. A technical replicate was used to verify sterility by the absence of growth after incubating the slide in LB broth for 48 h at 37°C. The slide was removed from the BSL-3 then processed and imaged as described above for the LC3 puncta assay. In this case the entire wells were manually searched for cells containing 5 or more LC3 positive phagosomes for bacteria infected monolayers (1026b, 576a, MSHR840 and MSHR435) and 10 or more puncta for autophagy controls (PBS and DMEM). The number of positive cells was divided by the capacity of the wells (2.5x10^5^ cells/well) to determine the percentage. The experiment was carried out in triplicate and the numbers represent the average of all three replicates with the error bars representing the SEM. One-way ANOVA was used to determine if the percentages from treated or infected monolayers and the DMEM negative control were significantly different.

### Human PBMC gene expression analysis in response to *Bp* LPS treatment

Fresh healthy human PBMCs were purchased from ZenBio (North Carolina, USA). The freshly received cells were seeded 1 mL per well in 12-well Corning CellBind plates with DMEM high glucose media with glutamine (HyClone) and 10% heat-inactivated FBS at a cell density of 1x10^6^ cell/ml. The plates were centrifuged at 400 x g for 8 min and incubated at 37°C in 5% CO_2_ overnight. The next day the cells in triplicate were treated with 10 ng/mL of LPS from the indicated strains or PBS for the non-treated controls. At the indicated time points supernatants were removed and frozen at -80°C for future cytokine analysis (below). Cells were treated in the same manner as RAW macrophages. RNA was isolated and cDNA was prepared the same as well. Human and innate adaptive immune response arrays from QIAGEN were used to analyze single replicates from PBMCs of two donors that were untreated, and treated with type A or type B LPS at the 24 h time point. Gene expression data was analyzed using the RT^2^ Profiler PCR Array Data Analysis version 3.5 software from SABiosciences.

### Cytokine secretion assay

Supernatants from 2 and 24 h treated PBMC from donor 1 and for comparison the 24 h time point from a second donor were analyzed for T_H_1 and T_H_2 associated inflammatory cytokines using the BioRad MAGPIX system from the three replicates in duplicate. The Human Ultrasensitive Cytokine Magnetic 10-Plex Panel assay (Thermo Fisher) was used to determine levels of GM-CSF, TNF-α, IL-2, IL-1β, IL-4, IFN-γ, IL-8, IL-10, IL-6, and IL-5 secreted by the human PBMCs in response to the LPS treatments. The assay was carried out on a Bio-Plex MAGPIX multiplex reader (Bio-Rad) using the Luminex xMAP technology. Supernatant was diluted 1:4 in DMEM and the assay was carried out according to the manufacturers recommendation. Target bead counts were set at a minimum of 100 for each cytokine and analyzed using the Bio-Plex Manager MP software. Generation of standard curves allowed extrapolation of secreted cytokine concentrations. Significance was determined by multiple one-way ANOVA comparisons using the Graphpad Prism software suite.

### Lipid A preparation

LPS samples were treated with 1% acetic acid with SDS for 1 h on a 100°C heat-block essentially as previously described [26]. Samples were lyophilized and resuspended in 100 μL of water using a sonicating water bath for 10 min. The SDS was removed by pelleting the sample with 500 μL of acidified ethanol (1% HCl in 100% ethanol) and centrifugation at 12,000 x g and 4°C for 10 min. The pellet was washed twice with non-acidified 100% ethanol and the sample was pelleted and dried. The 576a Type B LPS was resistant to the weak acid treatment above. Consequently, acid and SDS concentrations were increased in 2X increments and the time of heating was also increased. Samples were removed every 1 h to assess lipid A removal by running on SDS-PAGE gels followed by silver staining. It was found that 4% (w/v) SDS and 4% (w/v) acetic acid for 4 h was the minimum necessary to hydrolyze the O-antigen from the 576a Type B LPS. Similar observations with other *B*. *pseudomallei* LPS have been described [50] likely indicating heterogeneity in the core polysaccharides of the species. This sample was sent for MALDI-TOF/TOF at the Interdisciplinary Center for Biotechnology Research at the University of Florida. To make sure our modified preparation procedure was not affecting our lipid A preparation, a second procedure used to remove O-antigen from *Salmonella* LPS for MALDI analysis was implemented for isolating lipid A from an independent preparation of 576a LPS. This procedure [51] utilized 1 M acetic acid (6% w/v) treatment for 1.5–4 h at 100°C with purification as described above. Samples were dried and sent for MALDI-TOF/TOF analysis with nearly identical scans obtained from both independent preparations.

### MALDI-TOF/TOF and analysis

For MALDI-TOF/TOF analysis, dried samples were solubilized in 100 μl of a chloroform:methanol (2:1, v/v), and then prepared dilutions 1:10, 1:100, and 1:1000. To prepare the samples, 10 μL of each dilution were mixed with 10 μL of matrix (500 mM 2,5-dihydribenzoic, [51, 52] and 3 μL of ultrapure water, mixed and 0.5 μL of each preparation was spotted on a MALDI plate. Samples were dried out at room temperature. Mass spectrometry data was acquired in a 4700 MALDI-TOF/TOF analyzer (ABSciex, Framingham, MA, USA) with 4000 Explorer v3.0 software; and Nd:YAG (neodymium doped yttrium aluminum garnet) laser with 200-Hz sampling rate. The instrument was operated in the negative ion reflector mode, using a fixed laser intensity of 2500 to accumulate 1000 shots/spectrum, across the mass range of 1300–1900 Da. Acquired spectra was then analyzed with Data Explorer software v4.6. T2D files were converted to mzXML files and further analyzed on the open source mMass software. Three independently isolated LPS samples that underwent 2 different lipid A isolation protocols had their mass scans normalized and averaged for the spectrums presented.

## Results

### LPS purification and glycosyl composition

**Fig 1:**
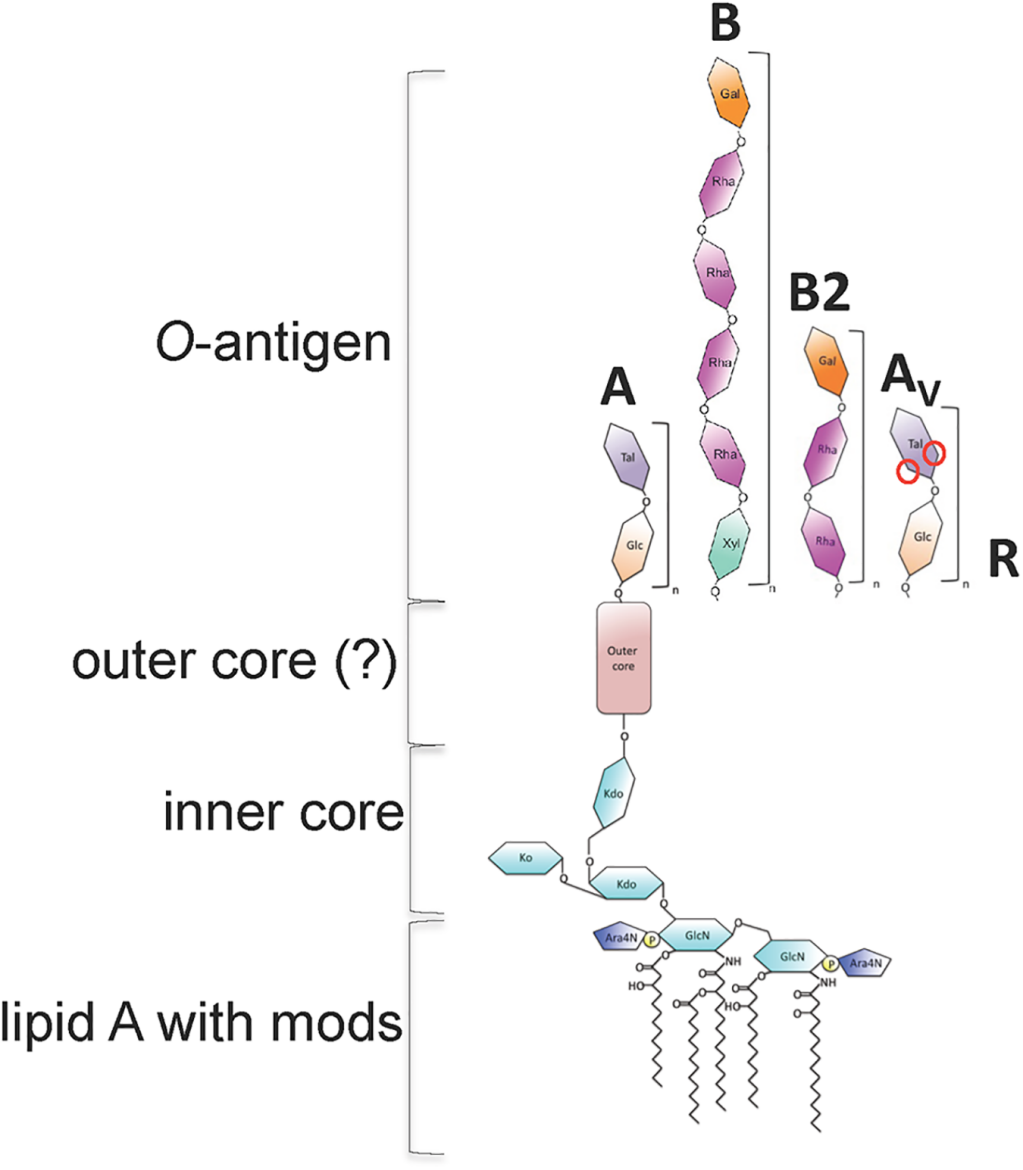
LPS types present in *B*. *pseudomallei* strains used in this study. The O-antigen structures shown here are based on previously published structures in the case of Type A and Type A_V_ and unpublished data in the cases of Type B and B2. Inner core is based on structures found in *Burkholderia cenocepacia* and lipid A is a composite of structures found in this work and others referenced in the text. *B*. *mallei* is used as an example of the Type A_V_ LPS and was not investigated in this study. Red circles indicate variable methylation and acetylation of the L-6dTal*p* residues of Type A_V_ LPS.

Previous reports showed that LPS from *Bp* strain KHW and K96243 were weak inducers of TNF-α while LPS from *Bt* was relatively strong, noting an inverse correlation between TNF-α levels and the two species’ pathogenicity [26]. At the commencement of our studies, the relationship between *Bp* LPS types and inflammation was unknown. To this end LPS was isolated from the Select Agent excluded strain Bp82, a derivative of the common LPS type A strain 1026b, which is considered a low virulence strain, and the LPS type B strain 576a, considered a high virulence strain. Strain 1026b was isolated form a non-fatal case of septicemic melioidosis and has an LD_50_ by intraperitoneal injection in BALB/c mice of 5.1x10^4^ CFU [53]. On the other hand, strain 576a was isolated from a fatal disseminated melioidosis case and has an LD_50_ of 80 CFU in the same murine melioidosis model [54]. For this and other structural characterization work, capsule deficient mutants of both of these strains were engineered by deleting genes of the capsule biosynthesis operon *wcb* [55, 56]. Western blot and silver staining indicated an absence of capsule, while LPS was unaffected. Type B2 LPS *Bp* strain MSHR840 and virulent rough strain MSHR435 were also included in the analyses [25]. Both strains were isolated from patients who died from CNS associated melioidosis in Australia. Capsule mutants of these strains were not generated because previous works determined that the presence of the CPS did not affect production of TNF-α, IL-6, or IL-10 [26]. During the phenol–water purification method employed in this study, type A LPS was found in the phenol phase, while Type B and B2 were primarily found in the aqueous phase. The relatively insoluble talose residues [57] present in type A LPS may decrease LPS solubility, similar to what was previously described for *Rhizobium loti* LPS [58]. Interestingly, LPS from the rough strain MSHR435 showed two distinct polysaccharides. A lower molecular weight polysaccharide at ~10 kD was found in the aqueous phase of the preparation while a higher molecular weight polysaccharide at ~30 kD was found in the phenol phase (S1 Fig). Similar results have been previously reported for *Yersinia enterolitica* LPS, where the O-antigen residues were shown to be responsible for LPS solubility [59]. After extensive dialysis, all phases were digested with DNase I, RNase H, then overnight with Proteinase K prior to analysis, precluding protein contamination of the sample. Silver stains capable of detecting 1 ng of material indicated highly pure preparations [60]. Glycosyl composition of the type B LPS from *Bp* strain 576a, type B2 LPS from *B*. *thailandensis* 82172 (used as a surrogate for MSHR840 since both strains are 90% homologous at the nucleotide level across the *O-*antigen biosynthetic operon), and the rough LPS from rough *Bp* strain MSHR435 are shown in Table 1. These strains have had their LPS types verified previously [38, 39]. Type B LPS was found to contain rhamnose, xylose, and galactose in a 4:1:1 ratio and other components of the core and lipid A. The type B2 LPS was found to contain rhamnose and galactose in a 2:1 ratio along with other components of the core and lipid A. The similar monosaccharide content, cross-reactivity of mAbs to both type B and B2, and similarity at the genetic level indicate partially conserved structures. The rough LPS shows a predominance of heptose core saccharides and glucose modifications of the core. A schematic showing the different LPS types present in the strains employed in this study is shown in Fig 1. Structures have been approximated based on glycosyl composition analysis.

**Table 1:** Glycosyl composition of type B, B2, and rough *Burkholderia* LPS.

	*B*. *pseudomallei* 576a Type B LPS		*B*. *thailandensis* 82172 Type B2 LPS		*B*. *pseudomallei* MSHR435 Type R LPS	
Glycosyl residue	Mass (μg)	Mol %	Mass (μg)	Mol %	Mass (μg)	Mol %
Ribose (Rib)	n.d.	-	4.6	2.7	n.d.	-
Arabinose (Ara)	n.d.	-	n.d.	-	n.d.	-
**Rhamnose (Rha)**	**81.3**	**43.4**	**93.8**	**50.9**	1.5	3.5
Fucose (Fuc)	n.d.	-	n.d.	-	n.d.	-
**Xylose (Xyl)**	**17.8**	**10.4**	**n.d.**	**-**	n.d.	-
Glucuronic Acid (GlcA)	n.d.	-	n.d.	-	n.d.	-
Galacturonic acid (GalA)	n.d.	-	n.d.	-	n.d.	-
Mannose (Man)	n.d.	-	1.1	0.5	1.1	2.4
**Galactose (Gal)**	**27.2**	**13.2**	**57.0**	**28.2**	1.5	3.3
Glucose (Glc)	11.3	5.5	8.0	3.9	10.9	24.0
N-Acetyl Galactosamine (GalNAc)	n.d.	-	n.d.	-	n.d.	-
N-Acetyl Glucosamine (GlcNAc)	n.d.	-	n.d.	-	n.d.	-
N-Acetyl Manosamine (ManNAc)	n.d.	-	n.d.	-	n.d.	-
Heptose	55.5	23.2	32.3	13.7	31.2	58.8
KDO	11.8	4.3	5.9	2.8	4.8	8.0
Total	204.9	100.0	202.7	100.0	51.0	100.0

### Nitric oxide production and TNF-α secretion in RAW264.7 macrophages

**Fig 2:**
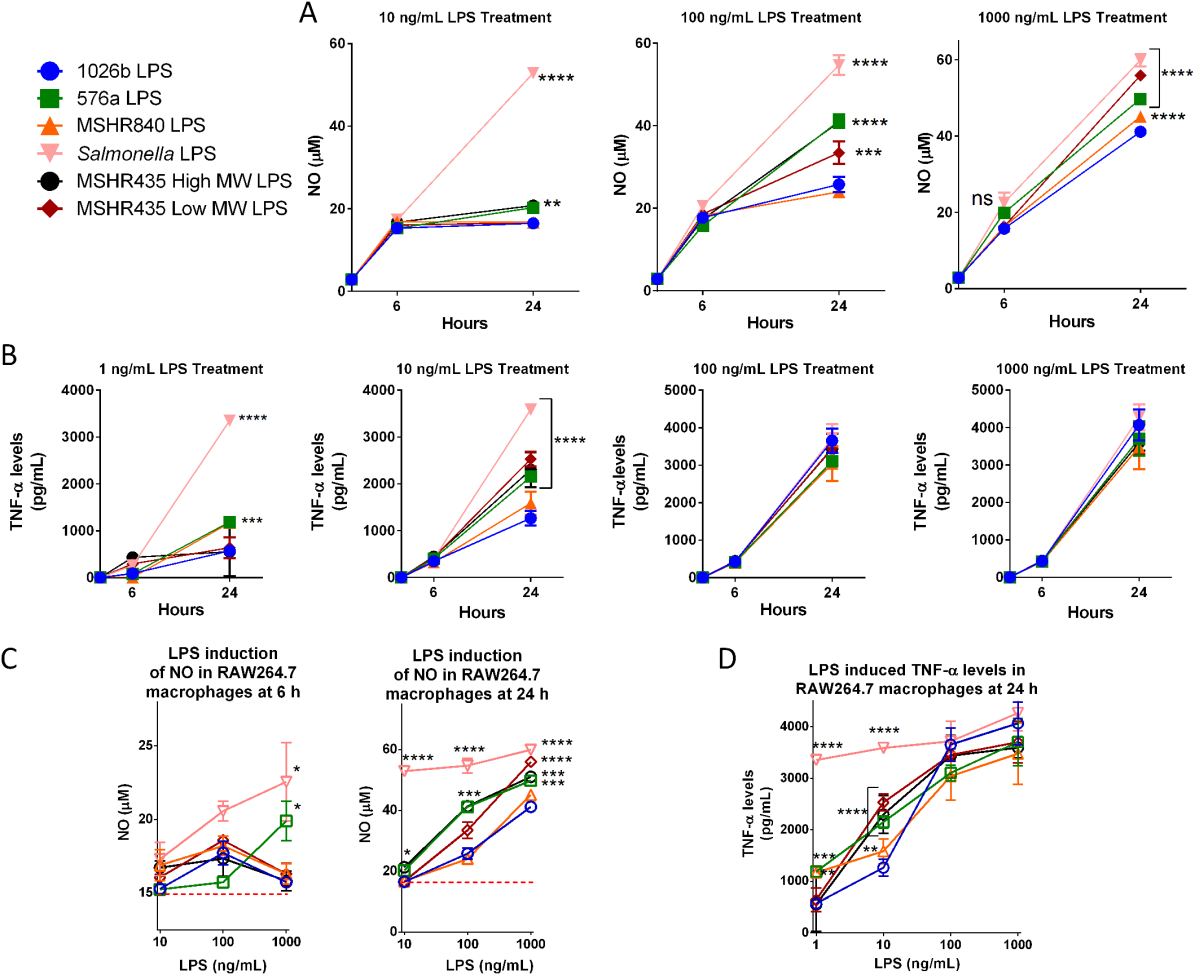
*Bp* LPS differentially induce inflammatory mediators NOS and TNF-α. RAW264.7 macrophages were treated with LPS isolated from the indicated strains. At the indicated time points iNOS (A) and TNF-α (B) secretion was measured (iNOS was assayed as a measure of total NO). (C), NO induction data in a dose-dependent format. (D), TNF-α secretion presented in a dose-dependent format. Red dashed lines indicated basal levels of NO. Differences between samples when compared to 1026b samples were significant as determined by two way ANOVA analysis, * = *p*<0.05, ** = *p<*0.005, *** = *p<*0.0005, **** = *p<*0.00005.

Nitric oxide (NO) production is highly induced during sepsis and endotoxic shock in mice and humans, resulting in increased vasodilation of vascular smooth muscle and organ failure. Purified preparations of the different LPS types were used to challenge RAW264.7 cells at varying concentrations starting with 10 ng/mL. It was found that 24 hours treatment with 100 ng/mL 1026b, 576a and MSHR840 LPS resulted in the largest differences in NO production in murine macrophages (Fig 2A). A similar trend was observed at the higher dose of 1,000 ng/mL. The 576a type B, both MSHR435 LPS, and *Salmonella* LPS generated significantly more NO release compared to the LPS from 1026b and MSHR840. Levels of NO induced at 6 h by all LPS types were low, with the *Salmonella* LPS control inducing the highest levels of NO (~22.5 μM) at the 1,000 ng/mL dose. At the same dose, 576a LPS was able to induce NO to a nearly equal level of 20 μM (Fig 2A). At 24 h post-treatment and all doses tested, the positive *Salmonella* LPS control showed maximum and sustained induction of NO at 60 μM. NO induction was low for all the samples at the lowest challenge dose of 10 ng/mL but levels in the 576a and the MSHR435 LMW LPS treated macrophages were significantly higher than those observed in 1026b LPS treated macrophages. At the next highest dose of 100 ng/mL, the 576a LPS and the higher MW LPS from MSHR435 induced NO levels were significantly higher than those obtained with LPS from 1026b and MSHR840, but still only half as much as NO production achieved with *Salmonella* LPS. At the maximal dose tested, 1,000 µg/mL, 576a LPS and both MSHR435 LPS were significantly better inducers of NO synthesis than 1026b and MSHR840 LPS. NO production is locally induced by the inflammatory mediator TNF-α, so the ability of the different LPS to induce TNF-α secretion was investigated. Supernatant from LPS treated RAW264.7 macrophages were used in a bioassay to determine TNF-α secretion levels over time and doses (Fig 2B). A LPS dose of 10 ng/mL provided the most significant differences in TNF-α induction with higher doses appearing to saturate TNF-α induction in this model. 576a LPS and both MSHR435 LPS induced significantly more TNF-α than 1026b LPS indicating a substantial difference in early phase innate immune signaling triggered by the different LPS samples. The studies indicate that significant TNF-α secretion levels occur earlier and at lower doses than NO production. This makes sense because NO formation is induced by TNF-α so TNF-α levels must first reach a critical threshold before commencement of the concomitant NO response. The ability of LPS to induce NO and TNF-α in a dose-dependent manner is more clearly presented in Fig 2C and 2D. The plateauing effect of more potent LPS treatments can be seen at higher doses.

### Innate and adaptive immunity qPCR arrays verify a more severe immune response to 576a type B LPS

**Fig 3:**
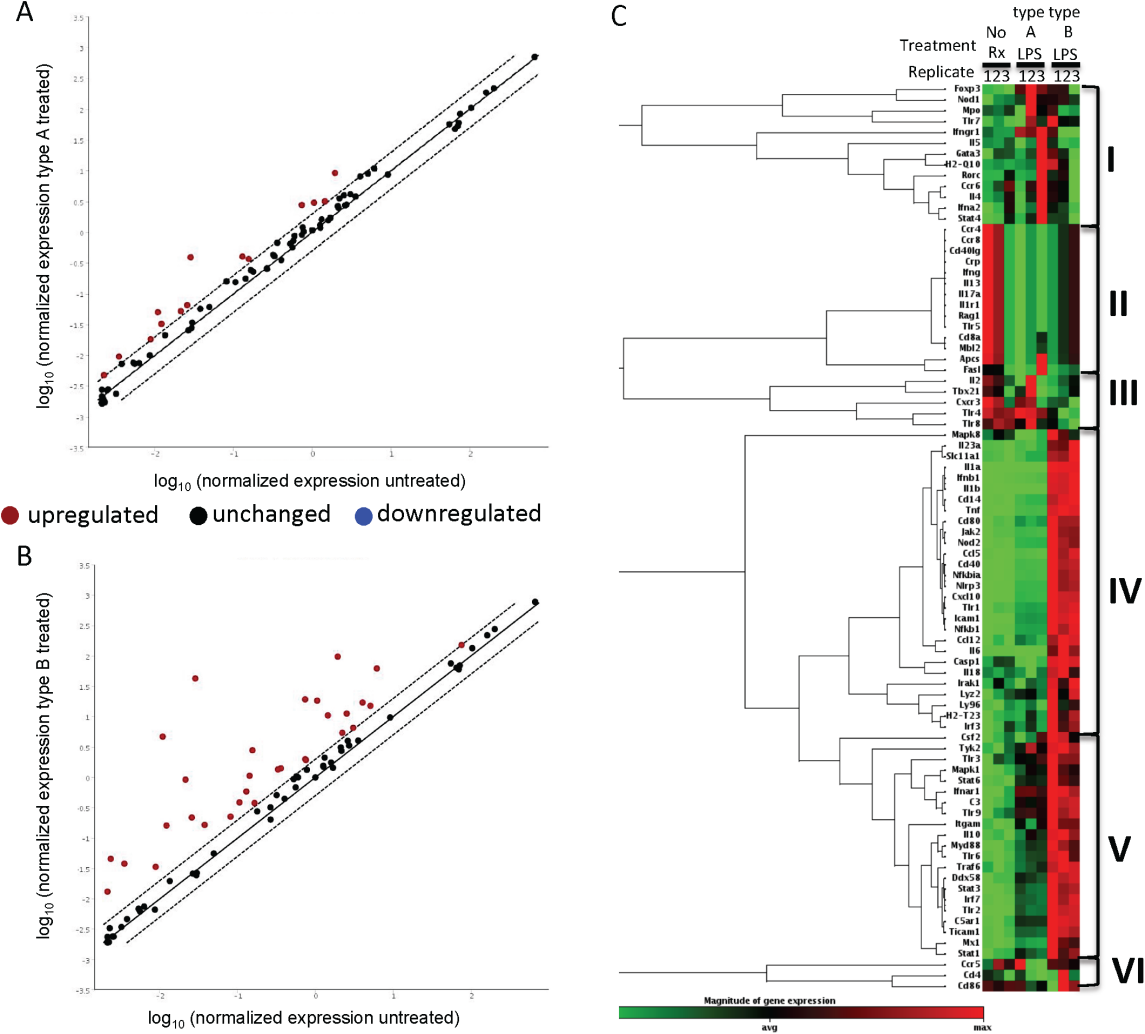
Dissimilar induction of innate and adaptive immunity by Type A and B LPS in RAW264.7 macrophages. Macrophages were treated for 2 h with either 10 ng/mL Type A or Type B LPS before isolation of total RNA. Gene expression profiles were assessed using innate and adaptive mouse RT^2^ Profiler qPCR arrays. Panels A and B show genes that are either upregulated more than 2-fold (red dots) unchanged (black dots) or downregulated more than 2-fold (blue dots) in response to macrophage treatment with Type A LPS (derived from 1026b) or Type B LPS (derived from 576a), when compared to untreated macrophages. C), Heat-map of all genes analyzed and grouped by expression trends. Genes with significantly different expression are listed in S1 Table.

The ability of the 576a Type B LPS to induce TNF-α and NO at levels significantly higher than the 1026b Type A LPS prompted a closer look at induction of innate and adaptive immune responses by the respective LPS types. Based on the previous experiments, an exposure regimen for 2 h with 10 ng/mL LPS was chosen as the optimal concentration and time at which to observe differential induction of innate and adaptive responses by Type A and Type B LPS in RAW264.7 macrophages. The employed array targeted 85 innate and adaptive immune genes plus 5 housekeeping genes and several sample controls. A comparison of the normalized gene expression of the gene targets in the 1026b LPS treated versus the untreated sample showed poor induction of innate and adaptive immune responses by the 1026b Type A LPS (red dots, Fig 3A). In contrast, the 576a type B LPS is capable of high induction of many innate and adaptive immune genes in mouse macrophages (red dots, Fig 3B). The heat map in Fig 3C compares the replicate samples and the gene expression from all genes on the array. The gene expression profiles can be grouped into six groups (I-VI). Group I genes show generally increased expression after treatment with 1026b Type A LPS. Genes associated with Th2 maturation such as Gata3, Il-4 and, Il-5 are upregulated in response to the 1026b Type A LPS but are not differentially expressed in Type B LPS treated and untreated samples. Nod1 and Tlr7 induction indicate a response targeting alternate PAMPs. Group II genes are those that are down regulated in both treatments compared to untreated macrophages. Ccr4 and Ccr8, found in this group, are receptors for important immunomodulators MCP-1, MIP-1, RANTES, TARC, macrophage derived chemokine and the chemoattractant CCL1, respectively. The cytokine genes Il13, Il17a, and Ifng (IFN-γ) are also in group II. Group III genes are down regulated in the 576a type B LPS treated samples. Tlr8 and the LPS receptor Tlr4 are in this subset. Group IV genes are uniquely upregulated in the 576a type B treated samples. Inflammation associated cytokine genes Il1a, Il1b, Il6, Il18, Il23a, and Tnf (TNF-α) along with Cd14 and Ly96 (MD-2), co-receptor genes for bacterial LPS, are in group IV. Downstream transcriptional regulators of the MyD88 dependent and MyD88 independent pathways, Nfkb1 (NFκ-B) and Irf3 respectively, are also present. The T-cell co-stimulatory genes, Cd40 and Cd80, were up regulated in the macrophages treated with 576a LPS, efficiently priming them for activation of T-cells. The trend of gene induction of group V is similar to group IV in terms of 576a type B LPS induction, but there is also a moderate induction in the samples treated with 1026b type A LPS. Notable genes in this group are the complement components C3 and C5ar1, and Tlr2, Tlr3, Tlr6, Tlr9, Myd88, Ifgam, Irf7, Traf6, Ticam1 (Trif), and the anti-inflammatory cytokine Il10. The trend for Group 6 is unclear and includes Ccr5, Cd4, and Cd86.

Genes that experienced a significant average induction greater than 2-fold induction in either the 1026b Type A LPS or 576a Type B LPS treated cells are listed in S1 Table. Acute phase cytokines, Il1a and Il1b, are induced a considerable 429.94 and 1490- fold, respectively, in the 576a Type B LPS treated macrophages compared to untreated samples and each ~100-fold higher than in the 1026b Type A LPS treated macrophages. Expression of Ifnb1 and Tnf in the Type B LPS treated sample was ~50-fold higher than in the untreated and ~10-fold higher than in the 1026b Type A treated sample. The immunomodulatory cytokine Il6 was also induced more than 20-fold higher in 576a Type B treated macrophage cells than in Type A treated cells. The monocyte and T-cell attracting chemokines, Cxcl10 and Ccl12, exhibited a 6.9-fold and 3.9-fold higher induction in Type B versus Type A treated macrophages. The antigen presenting cell co-stimulatory protein Cd40 was induced 7.6-fold higher in type B. Not surprisingly, inflammation was highly induced by the 576a Type A LPS across the panel of detected genes. The only gene that exhibited an induction higher in the 1026b Type A LPS treated macrophages was Foxp3 and only by 0.6-fold. The previous observations that Type A LPS is weakly immunogenic have been verified by this dataset and further support the notion that it is a weak inducer of innate and adaptive immunity. In contrast, the 576a Type B LPS is a more potent activator of innate and adaptive immune signaling.

### qPCR validation and pathway analysis

For array data verification, several targets from the cell surface interface, MyD88-dependent, and MyD88-independent pathways were chosen for qPCR array data verification. Gene expression from all samples was included in this analysis. At the cell interface, the independent qPCR results agreed in the moderately higher expression of Cd14 and Md2 (Ly96) in the 576a Type B LPS treated samples (S2A Fig). Tlr2 expression was not significantly higher, though the trend for higher expression in Type B, R, and *Salmonella* LPS treated samples was present. The calculated expression value for Tlr4 was not significantly different, in the Type A and B treated samples, but was slightly down regulated in Type B treated samples in agreement with the qPCR array. However, the independent validation revealed that Tlr4 was significantly down regulated ~10-fold in *Salmonella* LPS treated macrophages.

TLR4 can cause signal cascades through MyD88-dependent and independent pathways. A more detailed qPCR analysis of gene expression targets at the branch pathway after TLR4 was done for further clarification of genes induced in this pathway and for validation of the array data (S2B Fig). Ifng (interferon gamma) showed very low levels of induction in all samples. 576a Type B LPS was a potent inducer of Mcp1, Nfkb, Nos2, and Tnfa. The high MW Type R LPS from MSHR435 and the *Salmonella* LPS were also significant inducers of Mcp1, Nfkb, Nos2, and Tnfa. The 1026b Type A and MSHR840 Type B2 LPS were both weak inducers of the MyD88-dependent pathway. According to the qPCR array data there was high induction of type I interferon expression and regulatory expression associated with MyD88-independent pathways. Independent qPCR verified that the type I interferon gene IFNb was highly induced in the 576a type B, MSHR435 rough and *Salmonella* LPS treated samples (S2C Fig). IFNb was induced 30–40 fold by these LPS compared to the 1026b type A LPS. The MyD88-indepenent regulatory gene Irf3 and TRIF (Ticam1) were moderately induced in the 576a type B LPS treated macrophages.

S2D Fig, shows the many ways that LPS from infecting *Bp* could stimulate the various innate immune pathways. LPS shed from infecting bacteria can be bound to LPS binding protein (LBP), which is then transported from CD14 to the MD2/TLR4 complex allowing dimerization and signal transduction through the MyD88-depenent pathway resulting in TNF-α mediated inflammation. Signal transduction can also occur from the MD2/TLR4 dimer through TRAM and TRIF in the MyD88-independent pathway resulting in a type I interferon response. The intracellular nature of *Bp* means that MyD88-independent signaling can also occur by MD2/TLR4 transduction from phagocytic vesicles containing shed LPS or live bacteria. The type I interferon response can then modulate innate immune signaling during intracellular infection. Another means by which intracellular *Bp* modulates inflammation is by shedding LPS in the cell cytoplasm causing caspase-mediated cleavage of pro-IL1 or pro-IL18, thereby increasing inflammatory response to LPS. S2 Table shows a summary of the capabilities of the different LPS samples to induce the MyD88 dependent or independent pathways based on the qPCR data. The 1026b type A LPS is a poor inducer of both pathways. The type B, rough, and *Salmonella* LPS are significant inducers of both MyD88 dependent and independent pathways. Type B2 LPS was marginally more efficient than 1026b LPS at inducing MyD88 dependent pathway related genes but not the independent pathway related genes.

### Diverse LPS types and autophagy

**Fig 4:**
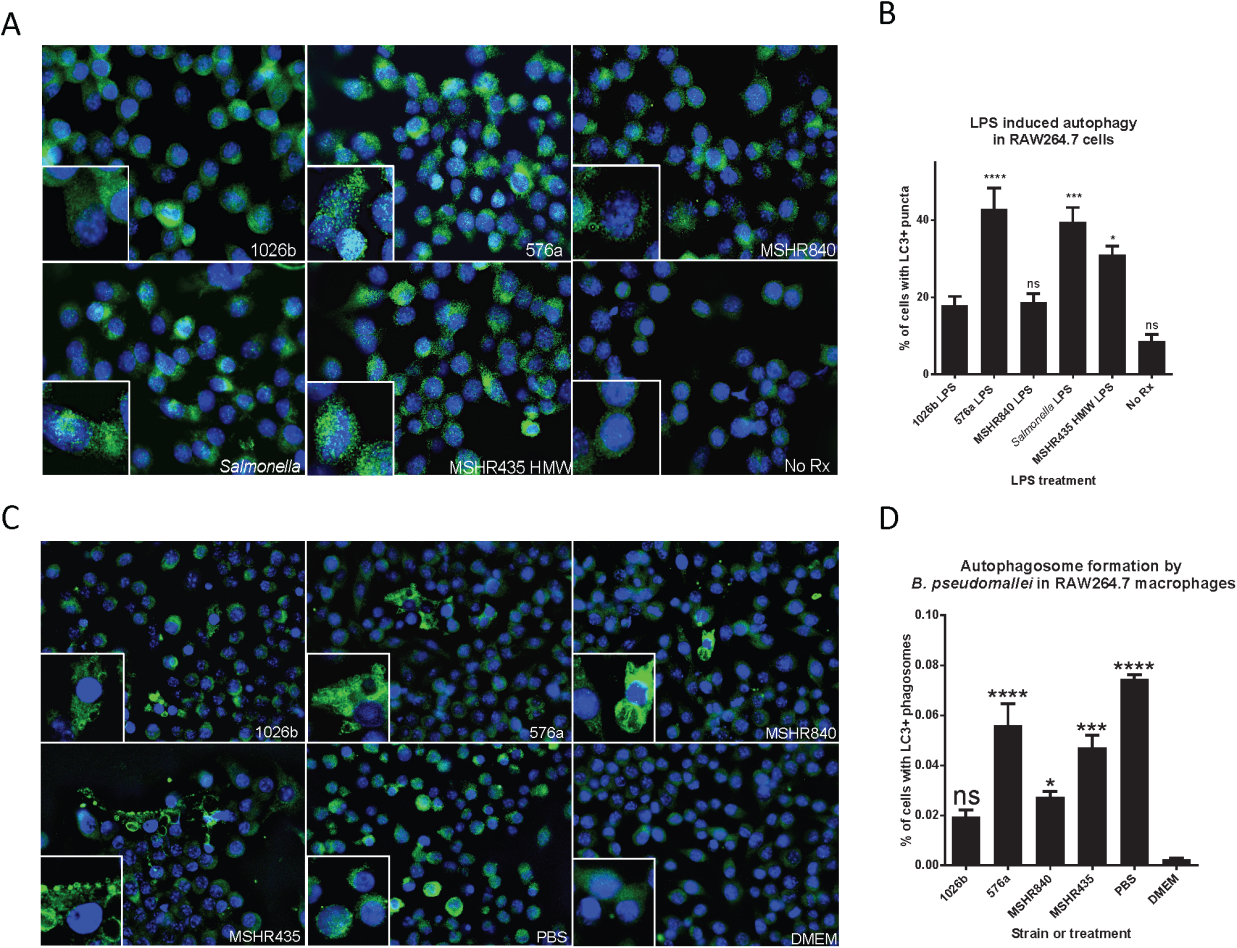
Differential induction of autophagy by purified *Bp* LPS and live bacteria. RAW264.7 cells were treated with LPS for 16 h and LC3-II+ puncta were observed (A) and LC3-II+ nuclei counted (B). The experiment was conducted in triplicate and 3 random fields of view were imaged for puncta analysis. Signal thresholding and particle analysis was used to correlate each nucleus with high levels of LC3 puncta. The Type B LPS from strain 576a and the rough strain MSHR435 HMW LPS were potent inducers of autophagy while type A from 1026b and type B2 from MSHR840 were weak inducers. RAW264.7 cells were infected with live *Bp* at an MOI of 10:1 for 6 h after which LC3-II+ phagosomes were observed (C) and percent of LC3-II+ cells counted (D). ns = not significant, * = *p*<0.05, *** = *p<*0.001, **** = *p<*0.0001.

Autophagy has been receiving significant attention as an innate immune mechanism of degrading intracellular bacteria. *Bp* has been shown to evade autophagy (for the most part) in RAW264.7 macrophages. The evasion is somewhat dependent on the T3SS effector BopA [16]. However, the ability to control intracellular *Bp* replication in human neutrophils was partially dependent on autophagic processes indicating killing of intracellular *Bp* by autophagy does occur [61]. Recently it has been shown that *Salmonella* LPS, through TLR4, can increase the amounts of autophagic vesicles present inside host-cell cytoplasm via the MyD88-independent signaling pathway [62]. The autophagy assay was carried out for 16 h to allow for adequate accumulation of autophagic vesicles. After 16 h of LPS treatment, macrophages were fixed and labeled for the autophagic vesicle marker LC3-I (Fig 4A). It was found that on average 576a, MSHR435, and *Salmonella* LPS treated monolayers contained the highest levels of fluorescent puncta. Some cells from the 1026b treated samples showed high levels of LC3 puncta, but this was not a monolayer-wide occurrence. The representative images show that all treated macrophages had higher levels of LC3 than the untreated (No Rx) samples. In the zoomed images, green LC3 puncta are visible in all samples but are much higher in the 576a, MSHR435, and *Salmonella* LPS treated monolayers. The fraction of cells with LC3 positive puncta are indicated in Fig 4B. Similar to the induction of other innate immune modulators described above, the LPS molecules derived from 576a, MSHR435, and *Salmonella* were potent inducers of the autophagy protein LC3. On average the 1026b and MSHR840 LPS were able to induce autophagy at least double that seen in the non-treated macrophages, just not significantly, and not to as high a level as the other treatments.

The ability of live *Bp* of different LPS types to induce autophagy was investigated by infecting the RAW264.7 cells for 6 h with live bacteria. It was found that monolayers infected with 576a and MSHR435 induced more cells to produce LC3 positive phagosomes and those cells that did had more present (Fig 4C). In Fig 4D, the amounts of LC3 positive phagosomes in 576a and MSHR435 infected monolayers were comparable to LC3 puncta formation in cells starved for 6 h in the presence of PBS (a well known inducer of LC3 positive puncta). 1026b was able to induce LC3 positive phagosomes in cells but both the number of cells with and the amount of LC3 positive phagosomes were less numerous and so not significantly different than the untreated DMEM control. Based on this data, LPS may be a contributor to autophagy induction during intracellular infection.

### Human PBMC gene expression and cytokine production assays

**Fig 5:**
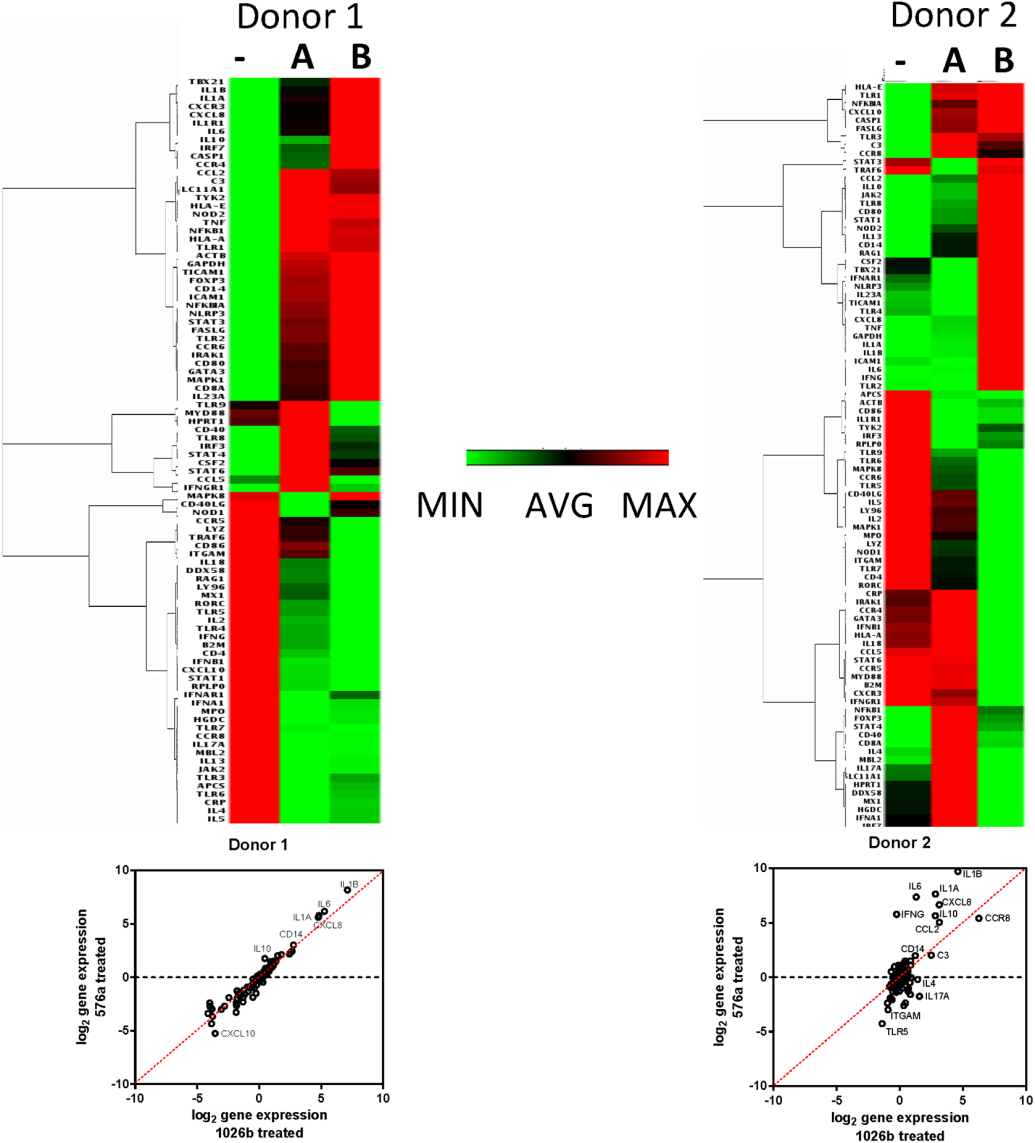
Dissimilar induction of innate and adaptive immunity by Type A and B LPS in human PBMCs. RT^2^ qPCR arrays were used to look at differences between Type A and B LPS in their ability to induce innate and adaptive immunity in healthy human derived PBMCs. Heat maps of expression levels of innate and adaptive immune targets from human PBMCs of two donors after 24 h treatment in mock treated (-), Type A LPS (from 1026b) treated (A), and Type B LPS (from 576a) treated (B) PBMCs. Red is the highest fold change in expression and green is the least. Log_2_ gene expression scatterplots are shown below the heat-maps.

For assessing human PBMC gene expression and cytokine production assays a 24 h treatment time point was chosen because macrophages only make up a small proportion of the total cells in PBMCs and we wanted to make sure our analysis measured gene induction environments after population wide response to the LPS. The human RT^2^ arrays showed that at 24 h post-treatment there were many differences in gene induction between Type A and B LPS treated PBMCs from both donors (Fig 5). What also is apparent is the variability between donors. Principal component analysis in S3 Fig shows donor and LPS type greatly affect the immune response to LPS treatment. PBMCs from donor 1 showed a similar response to both Type A and B LPS but gene induction levels were higher in response to Type B LPS treatment. PBMCs from donor 2 exhibited larger differences between the LPS Type A and B treatments. Several inflammatory markers showing induction in the mouse macrophages were also highly induced in response to Type B LPS treatment compared to Type A in both human PBMC donors. Il1a was induced ~2 and ~30 times higher in response to Type B LPS compared to Type A LPS in donor 1 and 2, respectively and ~100 times in the mouse macrophage experiment (Table 2). IL1β had a similar profile as Il1α. Il6 was induced ~2 and ~80 times higher in donors 1 and 2. For comparison, IL6 was induced 20 times more in Type B than Type A treated mouse macrophage experiments. IFN-γ and TNF-α fold changes were more ambiguous in this experiment so secreted cytokines were measured to understand the physiological relevance of the gene induction data.

**Table 2:** Gene expression ratios in LPS treated human PBMCs.

	Gene expression ratios (Sample vs no Rx)			
	Donor 1		Donor 2	
Gene	A Rx	B Rx	A Rx	B Rx
**C3**	5.73	4.81	5.65	4.04
**Ccl2**	6.35	5.49	8.81	32.83
**Cd14**	6.84	8.08	2.34	3.93
**Csf2**	1.13	1.07	0.63	1.41
**Cxcl8**	28.42	54.41	8.71	100.50
**Ifng**	0.66	0.59	0.84	54.46
**Il1a**	27.25	48.70	7.09	199.61
**Il1b**	140.28	288.01	24.40	838.76
**Il6**	38.75	71.99	2.46	165.05
**Il10**	1.37	3.39	7.05	49.78
**Tnf**	2.03	1.91	1.07	2.05
**Ccr5**	0.77	0.49	0.97	0.41
**Il2**	0.28	0.16	0.80	0.43
**Il4**	0.19	0.27	2.67	0.87
**Il5**	0.06	0.15	0.82	0.38

red, overexpression compared to untreated; blue, underexpression compared to untreated; black, expression within 2-fold change.

**Fig 6:**
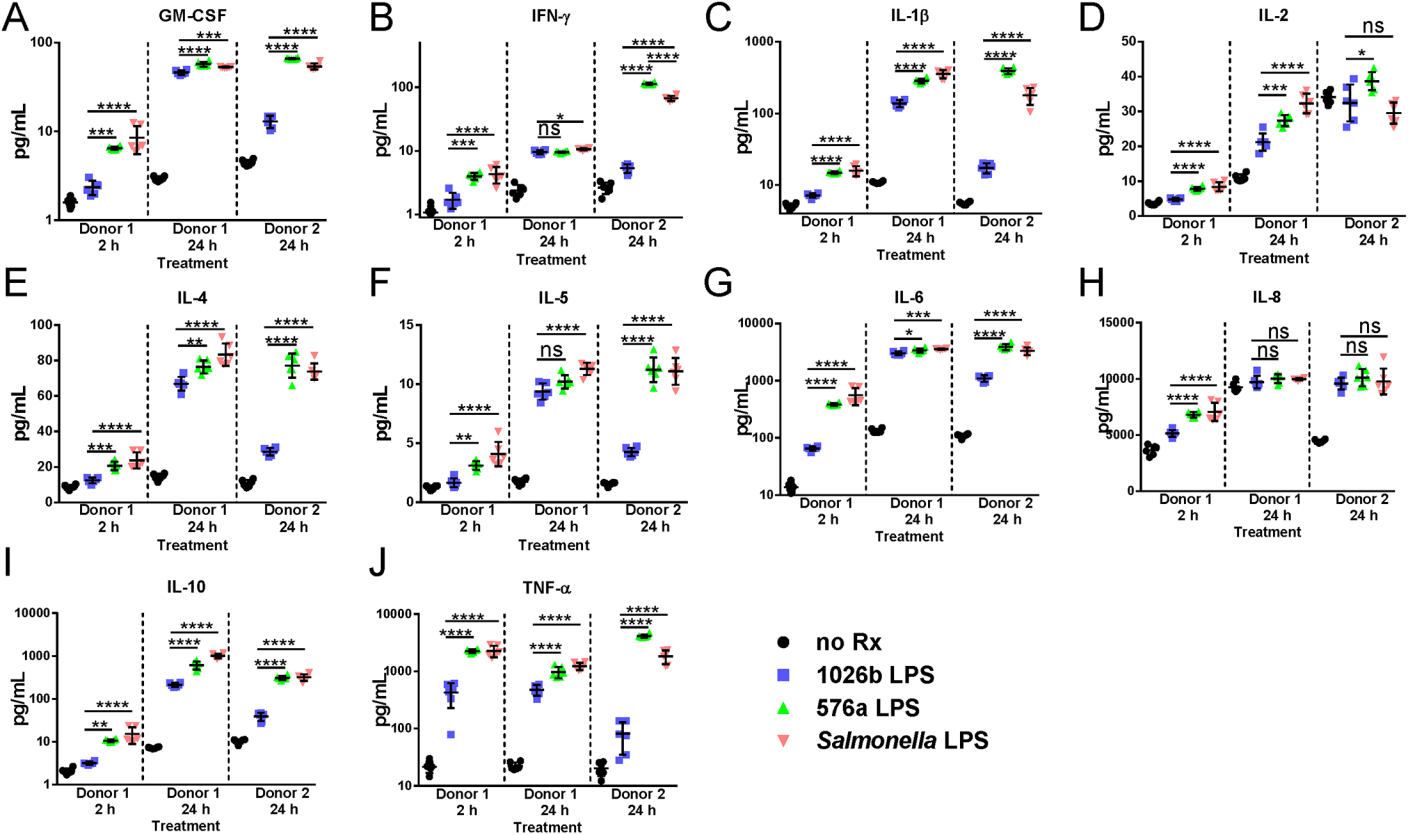
Cytokine secretion by human PBMCs in response to treatment with *B*. *pseudomallei* LPS. Levels of cytokines secreted by human PBMCs were measured at 2 and 24 h post LPS treatment for Donor 1 and 24 h post treatment from Donor 2. The cytokines analyzed are indicated above each plot with the sample origins indicated on the X-axis. Data for each sample set are divided by a dashed line. Mock treated negative control replicates are indicated by black circles, data obtained for Type A (1026b) LPS treatment are indicated by blue squares, and data obtained for Type B (576a) LPS treatment are indicated by green triangles. Salmon-colored triangles indicate data from positive control *S*. *minnesota* S-LPS treated samples. A), GM-CSF; B), IFN-γ; C), IL-1β; D), IL-2; E), IL-4; F), IL-5; G), IL-6; H), IL-8; I), IL-10; J), TNF-α. Scales are either linear or log depending on the cytokine for clarity. The means of all replicates and standard error of the means are indicated by error bars and lines. Differences between 576a compared to 1026b and *Salmonella* compared to 1026b treated were significant as determined by multiple one-way ANOVAs as shown above the datasets. ns = not significant, * = *p*<0.05, ** = *p*<0.005, *** = *p<*0.0005, **** = *p<*0.0001.

A set of 10 cytokines were selected to analyze the inflammatory response to *Bp* Type A LPS, Type B LPS, and *Salmonella* LPS. GM-CSF, TNF-α, IL-2, IL-1β, IL-4, IFN-γ, IL-8, IL-10, IL-6, and IL-5 measurements would provide a good snapshot of the cytokine environment at these time points (Fig 6A–6J). Initially it was found that gene expression at 2 h post-treatment was not significantly different between Type A and B LPS treated PBMCs but cytokine measurements at this time point reveal a different story. The Type B LPS induced cytokine secretion profile of all cytokines measured was significantly higher than Type A induced secretion and was more similar to *Salmonella* LPS treatment in donor 1 PBMCs. In PBMCs of the same donor at 24 h post-treatment cytokine levels were still significantly different for many cytokines measured the levels of which appeared to plateau. IFN-γ and IL-8 levels were no longer different in Type A and B treated PBMCs from donor 1. On the other hand, PBMCs from donor 2 showed highly significant differences between Type A and B induced cytokine secretion across all replicates at 24 h post-treatment. GM-CSF, TNF-α, IL-1β, IL-4, IFN-γ, IL-10, IL-6, and IL-5 had significance values of <0.0001 between Type A and B treated PBMCs. In fact, Type B LPS caused significantly elevated levels of TNF-α, IL-1β, IL-2 and IFN-γ secretion even when compared to *Salmonella* LPS treatment. Type B LPS was generally better at inducing cytokines associated with T_H_1 (TNF-α, IFN-γ, IL-2), T_H_2 (IL-4, IL-5), T_H_17 (IL-6) and Treg (IL-8, IL-10) T-cell responses and general pro-inflammatory mediators (GM-CSF and IL-1β).

### MALDI-TOF/TOF analysis of lipid A molecules and predicted structures

**Fig 7:**
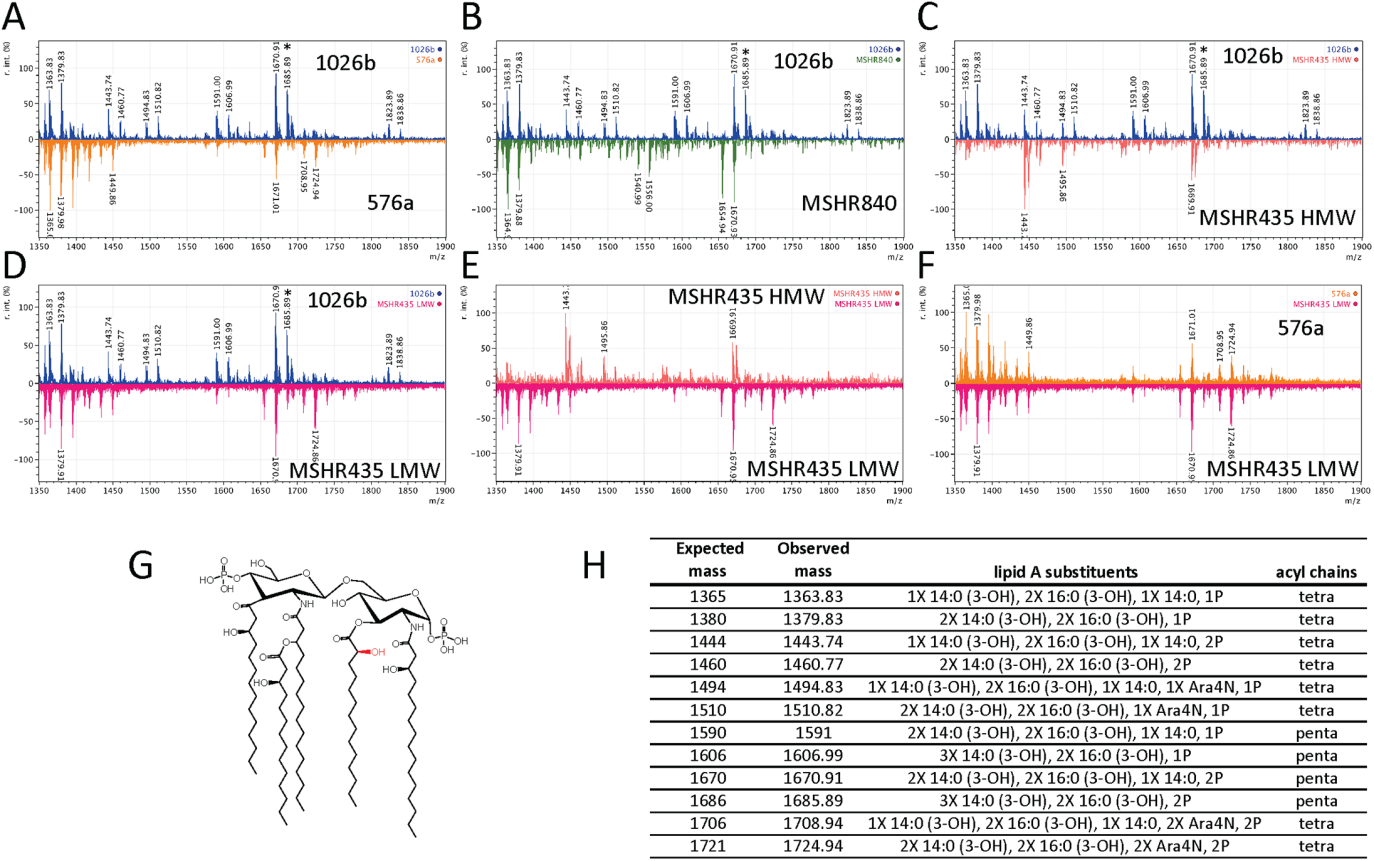
MALDI-TOF/TOF comparisons and predicted structures. The lipid A portion of the LPS from the indicated strains was purified and analyzed by MALDI-TOF/TOF. All lipid A scans are in the negative ion mode with mass from 1,000 to 2,000 Da on the x-axes and relative intensity on the y-axes. Scan traces are color-coded: 1026b scans are blue; 576a scans are orange, MSHR840 scans are green; MSHR435LMW scans are magenta; and MSHR435HMW scans are salmon. Panels A-F show comparisons of trace scans: A, 1026b (top) compared to 576a (bottom), B, 1026b (top) compared to MSHR840 (bottom), C, 576a (top) compared to MSHR840 (bottom), D, 1026b (top) compared to MSHR435LMW (bottom), E, MSHR435HMW (top) compared to MSHR435 LMW (bottom), and F, 576a (top) compared to MSHR435 LMW (bottom). Panel G shows the predicted structure of the predominant penta-acylated lipid A in the 1026b sample with the non-stoichiometric hydroxyl group not present in the 576a penta-acylated lipid A shown in red. The table in panel H, summarizes expected and observed masses of the indicated lipid A substituents and acyl chains.

It has been well studied that the lipid A component of the LPS is responsible for the molecule’s endotoxic and pro-inflammatory properties. The LPS types tested in the work presented in this study are hypothesized to possess different polysaccharide O-antigens that would engender vastly different antibody responses (Fig 1). However, the variance in innate immune induction by the different LPS types may be mostly contingent on diverse lipid A structures. MALDI-TOF was used to analyze the lipid A components of all LPS types tested in this study. Based on previously published data from *Bp* and *Bm* [26, 29], the MALDI-TOF/TOF spectra were analyzed in the 1300–1900 Da range for masses coinciding to tetra and penta-acylated lipid A molecules. Fig 7A shows the spectra observed by MALDI-TOF/TOF of the 1026b lipid A. Signals between 1364 and 1510 m/z indicate several types of variously hydroxylated, mono and diphosphorylated, and singly 4-amino arabinose (Ara4N) modified tetra-acylated lipid A species. Peaks at 1591 and 1606 indicate penta-acylated lipid A with a single phosphoryl group with and without an additional hydroxyl group respectively in agreement with previously published work [29]. Furthermore, peaks at 1670 and 1686 m/z coincide to the doubly phosphorylated (+79 m/z) structures just mentioned. Previously, the additional hydroxyl group detected at 1686 m/z (Fig 7, asterisk) was not found in the highly immunogenic *B*. *thailandensis* LPS [26], indicating the hydroxyl group may be responsible for the inability of penta-acylated 1026b *Bp* LPS to induce innate immunity. The crystal structure of the LPS/MD2/TLR4 complex suggests hydroxyl modification of the lipid A groups would interfere with the stability of the complex [63].

To examine this possibility, the 576a lipid A MALDI profile was compared to 1026b (Fig 7A). Mono-phosphorylated tetra-acylated lipid A were detected but with fewer additional modifications. A large amount of diphosphoryl penta-acylated lipid A species were detected at 1670 m/z but it is clear that the additional hydroxyl group found in 1026b at 1686 m/z is not present in the 576a sample. Mass peaks at 1708 and 1724 m/z indicated a diphosphorylated tetra-acylated lipid A with two Ara4N side group modifications. The lipid A from MSHR840 showed even less hydroxylation when compared to 1026b (Fig 7B) corresponding to less observed immunologic activity, while comparison of lipid A molecules from both MSHR435 LPS isolated show an absence of mass peaks at 1686 m/z and more similarities with the 576a lipid A (Fig 7C–7F) corresponding to increased levels of immunologic activity found in this work.

## Discussion

It is well known that some strains of *Bp* are more virulent than others [64, 65]. The root cause of this differential virulence is not entirely known. *In silico* predictions based on genomic diversity have tentatively identified some causes [66]. Being a highly immunogenic molecule, LPS is an ideal candidate for both induction and avoidance of host immunity. Several papers have focused on the LPS from type strains 1026b and K96243 but these strains are very similar and we hypothesized that variation in LPS O-antigen may also extend to variations in lipid A structures. We also believe that this key difference may have been partially responsible for the ability of the live-attenuated *Bp* 576a *ilvI* mutant vaccine 2D2 to protect BALB/c mice for up to 5 weeks from a 10^4^ CFU challenge by a virulent strain [54]. The observed differences with different LPS types may also be responsible for the efficient CD4+ T-cell mediated immune response observed in C57BL/6 mice [67]. In this study we attempted to understand if the innate immune signaling in response to purified LPS was dissimilar between *Bp* strains of different LPS types and why.

It was found that increasing incubation time to 6 h and beyond or increasing LPS concentration beyond 10 ng/mL saturated the model and could mask differences in TNF-α induced cytotoxicity and secretion. TNF-α secretion was still significantly higher in the 576a treated macrophages. Many studies observe LPS induction of innate immunity at 24 h and with higher concentrations of LPS or both. It is conceivable that when using such an approach differences in biologically relevant signaling between different LPS types may be overlooked if assays are allowed to progress for too long or are carried out with excessive concentrations of LPS. Although levels of secreted pro-inflammatory mediators like TNF-α and iNOS continue to increase for up to 24 h, initial stages of gene induction set the stage for pathogen response and cellular infiltration.

In this study, the gene expression profiling was focused on the early time points to detect initial responses to LPS in macrophages. The assays focused on LPS at a concentration of 10 ng/mL and its ability to induce gene expression of immunomodulators early in the signaling cascade. Previous works described *Bp* LPS as a weak inducer of innate immunity and this notion agreed with our findings with 1026b and MSHR840 LPS. In contrast, the Type B LPS from strain 576a was an exceptional inducer of innate and T-cell response genes. The data is in agreement with previous works clustering the gene induction profile of RAW264.7 macrophages infected by *Bp* 576a bacteria at 4 h post infection (hpi) with exposure to highly immunogenic hexa-acylated *E*. *coli* LPS at 4 and 8 hpi [68]. It is worth noting that in the same study the gene induction profile of RAW264.7 macrophages infected by *Bp* 576a bacteria at 8 hpi clustered with the profile of *Bp* 1026b and K96243 infected RAW264.7 macrophages at 8 hpi, in further support that the receptors become saturated over time. Systemic induction of innate and adaptive mediators was observed by the 576a Type B LPS; illustrated by increased type I interferon responses, and induction of both MyD88-dependent and independent responses. The resulting gene induction of pro-inflammatory cytokines, macrophage and T-cell specific chemokines, and T-cell co-regulatory molecules suggests that the 576a Type B LPS would bridge the gap between the innate and adaptive immune activation typically seen with LPS similar to that of 1026b.

The anti-viral action of type I interferon in viral infections has been well characterized. Evidence shows that the type I interferon response can be both detrimental to the host by suppressing innate immune responses to infecting bacteria, in the examples of *Listeria monocytogenes* [69] and *Mycobacterium tuberculosis* [70], and supportive of bacterial clearance, in the examples of *Legionella pneumophila* [71] and *Salmonella typhimurium* [72]. Increased levels of type I interferon are also associated with more virulent strains of *M*. *tuberculosis* and *L*. *monocytogenes*. The induction of type I interferon in macrophages and dendritic cells in response to bacterial infections has been canonically traced to MyD88-independent signaling via TLR4 responses to LPS. The type I interferon response to or effect on *Bp* infection has not been characterized but it may play a role in driving inflammation.

Another aspect of LPS based effects on immunopathogenesis is signaling through TLR4 and its activation of autophagy via the MyD88-independent signaling pathway. *Salmonella* LPS at 100 ng/mL has been shown to cause signaling through TRAM and TRIF, presumably through endosomal compartments, resulting in maximal autophagy induction at 16 h post-exposure and enhanced co-localization of *M*. *tuberculosis* with autophagosomes [62]. In this study 10 ng/mL LPS was used for consistency in approach but differences in autophagy induction were still observable. Whether or not the live intracellular *Bp* induce different levels of autophagic vesicles was unknown so RAW264.7 macrophages were infected with the live LPS source strains used in the above assay. The data mirrored the purified LPS assay, specifically that MSHR435 and 576a induced more autophagosomes in more cells than 1026b or MSHR840. Cells were untreated or starved in PBS as LC3II+ vesicle positive and negative controls, respectively. It is known that *Bp* evades autophagy. In response to increased autophagy, *Bp* may by upregulate evasion mechanisms, such as the *Bsa* and T3SS effector BopA. The bacterial response to increased autophagy during infection is the subject of future study, the main point being that the diversity of *Bp* LPS causes significantly different innate immune responses that correlate with intracellular presence of the bacteria.

Gene induction by the different LPS types was verified in healthy human donated PBMCs. Large differences in gene expression profiles in the PBMCs treated with Type A and B LPS were observed in cells from two donors. Amongst donors, there were several similar genes induced but there was also heterogeneity between donors, highlighting the importance the population diversity plays in immunological response and susceptibility to bacterial infections. Many pro-inflammatory cytokine expression levels such as Il1β, Tnfa, and Ifng showed variation between donors while others (Il6 and Il8) did not. The production of these mediators is constantly in flux. Some individuals may have different resting states of expression. For example, the second donor had a high resting level of Stat3 expression that would possibly lead to a delay in production of pro-inflammatory mediators IL-1β, TNF-α, and IFN-γ. The physiologic result of gene induction data was explored by measuring important inflammatory cytokines secreted by PBMCs from two donors in response to treatment with various LPS. Type B LPS from strain 576a was able to generate a significantly stronger immune response in human cells than Type A LPS from 1026b. This difference in LPS innate immune induction may help explain the significant protection live-attenuated *Bp* 576a strain 2D2 can convey upon BALB/c mice [54, 73] when compared to experiments utilizing other bacterial strain backgrounds for vaccination/challenge experiments [10]. Because the protection is so significant, strain 576a has been proposed as a positive control for vaccination experiments by the Steering Group on Melioidosis Vaccine Development [74].

The MALDI-TOF/TOF analysis of the different lipid As show that 1026b Type A LPS preparations contain a mixture of tetra- and penta-acylated lipid A with variable phosphorylation and hydroxylation; similar mass peaks as previously published [29, 52]. However, the Type B LPS from 576a contained less of the variably modified lipid A and nearly all the penta-acylated lipid A lacked the additional hydroxylation previously identified in other *Bp* lipid A molecules, suggesting the lipid A from 576a is structurally similar to that of *Bt*. It becomes clearer that *Bp* possesses many mechanisms to modify the potent immunologic LPS. All strains showed an ability to modify the parental penta-acylated diphosphorylated lipid A by removing acyl chains, phosphate groups, and masking of phosphoryl groups through Ara4N modification. Differential hydroxylation now appears to be an ability that only some *Bp* use to evade host immunity and is not found or differentially regulated amongst other strains. *Francisella tularensis* has also been shown to modify lipid A by removal of acyl chains and phosphates after transport to the outer membrane [75, 76], while *Yersinia pestis* begins producing tri- and tetra-acylated LPS when grown at 37°C [77, 78]. Recently, systematic characterization of *E*. *coli* LPS has revealed the additive effect of the functional modifications listed above on reduction of lipid A immunogenicity [79]. The downstream impact of lipid A modifications on *Bp* immunopathogenesis *in vivo* remains to be characterized.

Future work includes investigating the contribution of lipid A structure to binding to host proteins involved in innate immune signaling; such as MD2, CD14, and LBP and whether or not O-antigen structures are involved. These finding would also shed light on interactions between LPS and host-proteins during sepsis. Of specific interest is to ascertain if the lipid A diversity described herein is unique to strain 576a or is present in other Type B LPS expressing strains and other high-virulence strains from Africa or Southeast Asia. More complex *in vivo* model systems, such as the mouse model of inhalation melioidosis are required to fully characterize the downstream effects of the differential gene expression and cytokine secretion observed in this study. As more genetic diversity within *Bp* is discovered, the intricacies of *Bp* infection become more complex, increasing the depth and breadth of our understanding of bacterial immunopathogenesis.

## Supporting information

S1 Methods (DOCX)

S1 Table Macrophage genes upregulated in response to LPS treatment. (PDF)

S2 Table Innate immune pathway induction by the different LPS types. (PDF)

S1 Fig Silver stain gel of rough LPS isolated from *Bp* MSHR435. (PDF)

S2 Fig qPCR analysis of LPS innate immune signaling in RAW264.7 macrophages. (PDF)

S3 Fig PCA analysis of LPS induced responses in human PBMCs. (PDF)
