# Supplementary material for: Structural diversity of Burkholderia pseudomallei lipopolysaccharides affects innate immune signaling
Source: PLoS Negl Trop Dis. 2017 Apr 28;11(4):e0005571. doi: 10.1371/journal.pntd.0005571 (PMC5425228; doi:10.1371/journal.pntd.0005571)
Supplement: S1 Methods — (DOCX) [file pntd.0005571.s001.docx]

**Structural diversity of *Burkholderia pseudomallei* lipopolysaccharides affects innate immune signaling**

Michael H. Norris, Herbert P. Schweizer and Apichai Tuanyok

**Supporting Information**

**S1 Methods**

**Construction of the Bp82 Δ(*wcbR-A*) capsular polysaccharide mutant**

The entire operon required for capsular polysaccharide (CPS-I) biosynthesis was deleted by allelic recombination using previously published methods [1]. Briefly, an 813 bp fragment from the *wcbR* gene and an 845 bp fragment from the *wcbA* gene located 23,098 bp downstream in the *Bp* 1026b genome were generated by PCR and combined with an *FRT-ble-FRT* cassette PCR product. The Δ(*wcbR****-****A*::*FRT-ble*-*FRT*) fragment was created using splicing by overlap extension PCR and TA cloned into the pGEM-T Easy vector (Promega, Madison, WI). After verification of the presence of the correct construct, an EcoRI fragment was subcloned into EcoRI-digested pExKm5 [2] to create the gene replacement vector pExKm5-Δ*wcbR****-****A*::*FRT-ble*. Allelic replacement and Flp excision was carried out as previously described [1-3] and the presence of the Δ(*wcbR****-****A*::*FRT*) mutant allele was verified by PCR and sequencing. Loss of capsule production was verified by Western blot using mAbs to the *Bp* CPS [4].

**Creation of the 576a Δ*wcbB* mutant**

The *Bp* 576a Δ*wcbB* mutant was created by using previously published methods [1]. Briefly, the *wcbB* gene from 576a was amplified using PCR with oligonucleotides containing NotI sites and cloned into pGEM-T Easy. The plasmid was double digested with NheI+SalI then blunt ended and ligated. This resulted in deletion of 878 bp of the *wcbB* coding sequence. The ∆*wcbB* fragment was removed from the pGEM-T Easy vector by digesting with NotI and ligated into the same site of pExKm5 to produce pExKm5-∆*wcbB_Bp_*. The plasmid was introduced into 576a via electroporation and merodiploids identified using on LB medium containing 1,000 μg/mL kanamycin (Km) and 50 μg/mL 5-bromo-4-chloro-3-indolyl-β-D-glucuronic acid (X-Gluc). Km resistant and blue colored colonies were streaked on LB+15% (w/v) sucrose plates for merodiploid resolution. Colonies were screened on X-Gluc for loss of the plasmid backbone then PCR verified for the chromosomal deletion of *wcbB*. Loss of capsule production was verified by Western blot using mAbs to the *Bp* CPS [4].

**SDS-PAGE, silver staining, and Western blots**

Crude LPS sample preparation was carried out as previously [5] described. Crude and purified LPS samples were mixed with 2X LPS lysis/loading buffer prior to running on a gel. SDS-PAGE was carried out using homemade polyacrylamide gels with a 12% resolving gel and a 4% stacking gel. Silver staining of SDS-PAGE gels was carried out using the Pierce™ Silver Stain Kit according to the manufacturers instructions. Colorimetric Western blots were performed by semi-dry electroblotting of SDS-PAGE run gels onto methanol soaked Immobilon P^SQ^ PVDF membranes from Millipore™ or Odyssey nitrocellulose membranes from LI-COR. Blots were washed with 1xPBS, blocked with 1% skim milk in PBS and detected with 1-Step Ultra TMB-Blotting Solution (Thermo Scientific) following manufacturer’s instructions.

**qPCR and pathway validation**

Primers from the NIH mouse primer depot were used in SYBR green-based qPCR assays to validate pathway data obtained from the RT^2^ arrays. RNA was isolated from an independent experiment and cDNA was synthesized the same as above and qPCR was carried out the same. Efficiency determination and melt-curve analysis were carried out prior to qPCR. β-actin and GAPDH were chosen as house-keeping genes. qPCR pathway validation was done in technical and biological triplicate with a total of 9 Ct values for each sample. Ct and efficiency data were exported and analyzed using the REST software suite that takes into account the efficiencies of targets and house keeping genes [6]. The associated errors are propagated, giving higher stringency to gene expression significance determination when comparing two or more genes.

**Principle component analysis of hPBMC gene expression data**

Gene expression data were log2 transformed and analyzed with the Strata 13 software suite. Multivariate analysis shows that both host variability and LPS type were the major components effecting gene expression in the two human donor PBMCs populations.

**References**

1. Choi K-H, Mima T, Casart Y, Rholl D, Kumar A, Beacham IR, et al. Genetic tools for select-agent-compliant manipulation of *Burkholderia pseudomallei*. Appl Environ Microbiol. 2008;74(4):1064-75.

2. Lopez CM, Rholl DA, Trunck LA, Schweizer HP. Versatile dual-technology system for markerless allele replacement in *Burkholderia pseudomallei*. Appl Environ Microbiol. 2009;75(20):6496-503.

3. Propst KL, Mima T, Choi K-H, Dow SW, Schweizer HP. A *Burkholderia pseudomallei* Δ*purM* mutant is avirulent in immunocompetent and immunodeficient animals: candidate strain for exclusion from select-agent lists. Infect Immun. 2010;78(7):3136-43.

4. Nuti DE, Crump RB, Dwi Handayani F, Chantratita N, Peacock SJ, Bowen R, et al. Identification of circulating bacterial antigens by in vivo microbial antigen discovery. mBio. 2011;2(4).

5. Tuanyok A, Stone JK, Mayo M, Kaestli M, Gruendike J, Georgia S, et al. The genetic and molecular basis of O-antigenic diversity in *Burkholderia pseudomallei* lipopolysaccharide. PLoS Negl Trop Dis. 2012;6(1):e1453.

6. Pfaffl MW, Horgan GW, Dempfle L. Relative expression software tool (REST) for group-wise comparison and statistical analysis of relative expression results in real-time PCR. Nucleic Acids Res. 2002;30(9):e36.
