# Supplementary material for: Structural diversity of Burkholderia pseudomallei lipopolysaccharides affects innate immune signaling
Source: PLoS Negl Trop Dis. 2017 Apr 28;11(4):e0005571. doi: 10.1371/journal.pntd.0005571 (PMC5425228; doi:10.1371/journal.pntd.0005571)
Supplement: S1 Table — (PDF) [file pntd.0005571.s002.pdf]

	Genes upregulated in resposne to LPS treatment			
	Type A treated		Type B treated	
Gene	Fold Regulation	p-value	Fold Regulation	p-value
C3	1.94	–	2.78	0.0001
C5ar1	1.78	–	2.97	0.00008
Ccl12	2.71	–	10.81	0.00174
Ccl5	1.24	–	7.47	0.000056
Cd14	1.11	–	2.02	0.000012
Cd40	2.38	0.000057	18.03	0.000024
Cd80	1.46	–	3.67	0.000133
Csf2	1.51	–	4.37	0.015724
Cxcl10	3.78	0.000068	26.12	0.000003
Ddx58	1.6	–	2.57	0.000032
Foxp3	2.1	0.002029	1.45	–
Icam1	2.19	0.000003	7.26	0.000001
Ifnb1	2.44	0.029364	42.89	0.000001
Il10	2.06	0.023755	3.79	0.00023
Il1a	4.59	0.002784	429.94	0
Il1b	13.67	0.000011	1489.51	0.000001
Il23a	1.3	–	6.18	0.000926
Il6	-1.35	–	19.4	0.000565
Irf7	1.35	–	2.15	0.000057
Jak2	1.23	–	4.11	0.000308
Mx1	2.53	0.001237	8.47	0.002399
Myd88	1.61	–	2.45	0.001482
Nfkb1	1.58	–	4.47	0.000039
Nfkbia	1.76	–	10.14	0.000092
Nlrp3	2.88	0.000002	17.52	0.000112
Nod2	2.66	0.00101	13.2	0.000221
Ticam1	1.89	–	3.93	0.0000407
Tlr1	1.23	–	2.7	0.000009
Tlr2	2	–	4.25	0.000004
Tlr6	1.47	–	2.3	0.001377
Tlr9	3.1	0.008472	4.53	0.001684
Tnf	4.77	0.000023	50.56	0

S1 Table. Macrophage genes upregulated in response to LPS treatment.
