# Supplementary material for: Structural diversity of Burkholderia pseudomallei lipopolysaccharides affects innate immune signaling
Source: PLoS Negl Trop Dis. 2017 Apr 28;11(4):e0005571. doi: 10.1371/journal.pntd.0005571 (PMC5425228; doi:10.1371/journal.pntd.0005571)
Supplement: S2 Table — (PDF) [file pntd.0005571.s003.pdf]

**LPS sample**	**MyD88 dependent pathway induction**	**MyD88 independent pathway induction**
Type A	NS	NS
Type B	***	***
Type B2	**	NS
Rough	***	***
Salmonella	***	***

NS, not significant; **, $p<0.005$; ***, $p<0.0005$

S2 Table. Innate immune pathway induction by the different LPS types.
