# Supplementary figures and images for: Structural diversity of Burkholderia pseudomallei lipopolysaccharides affects innate immune signaling
Source: PLoS Negl Trop Dis. 2017 Apr 28;11(4):e0005571. doi: 10.1371/journal.pntd.0005571 (PMC5425228; doi:10.1371/journal.pntd.0005571)

# MSHR435 type R LPS

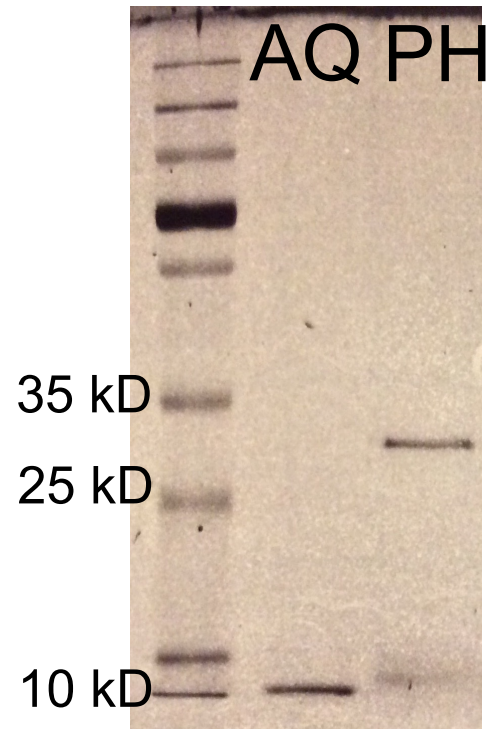

S1 Fig. Silver stain gel of rough LPS isolated from *Bp\_MSHR435*

Supplement: S1 Fig — (PDF) [file pntd.0005571.s004.pdf]

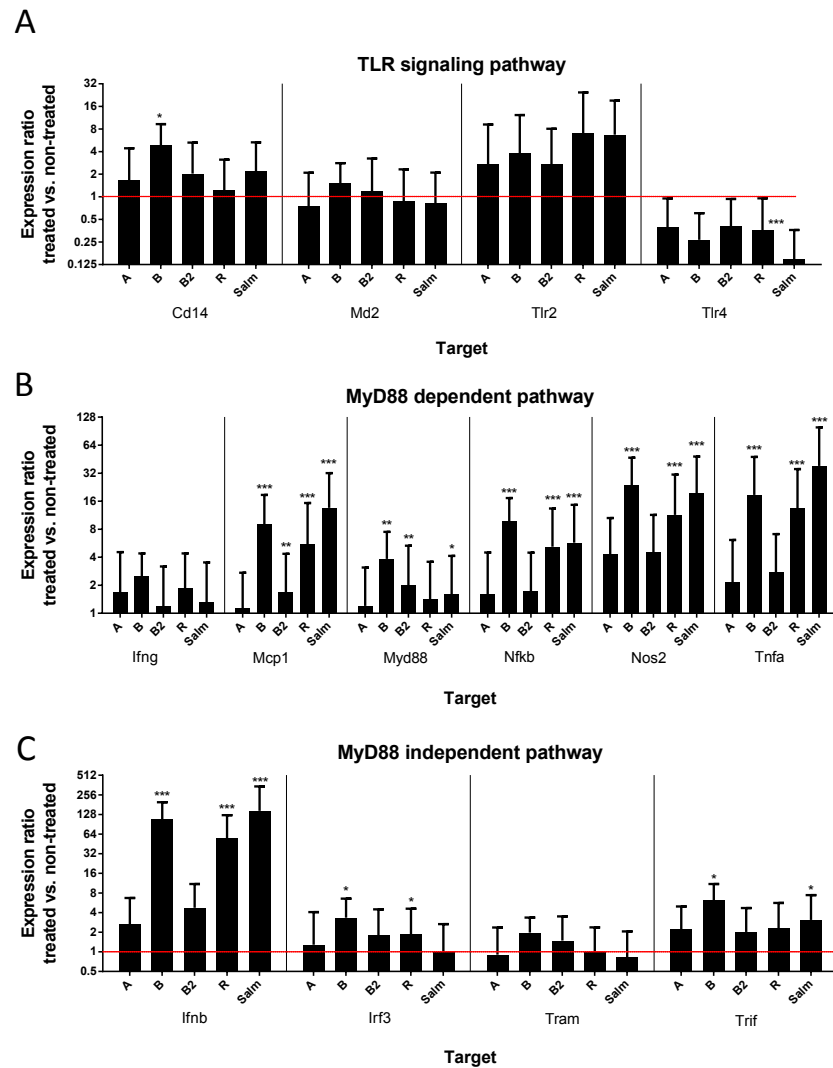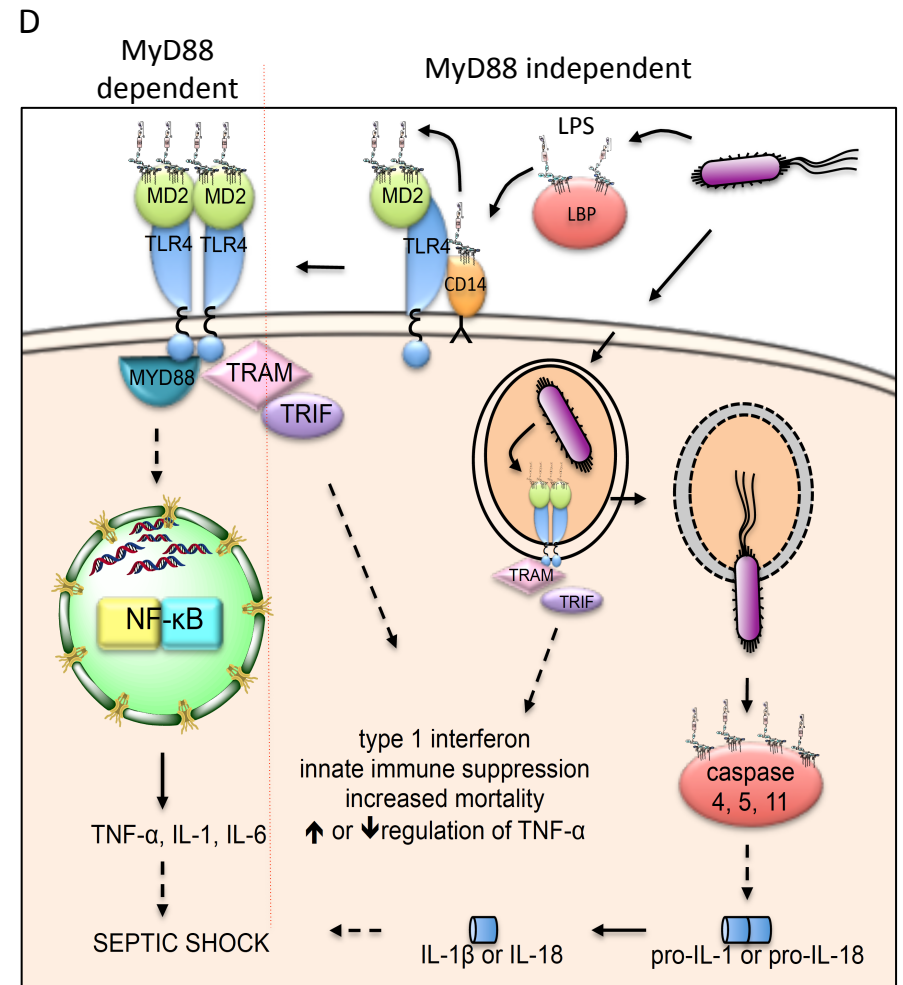

**S2 Fig. qPCR analysis of LPS innate immune signaling in RAW264.7 macrophages.**

Supplement: S2 Fig — (PDF) [file pntd.0005571.s005.pdf]

# PCA analysis of hPBMC treated with LPS

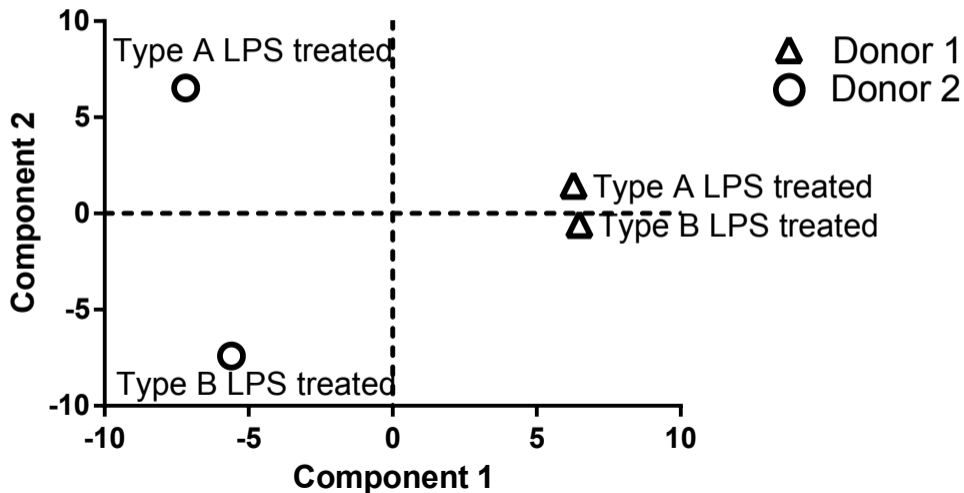

Supplement: S3 Fig — (PDF) [file pntd.0005571.s006.pdf]
